# Directional Reconstruction of Spent Lithium Cobalt Oxide by Microwave Plasma: Efficient Oxygen Evolution Catalyst from Closed‑Loop Recovered Resources

**DOI:** 10.1007/s40820-026-02164-1

**Published:** 2026-04-13

**Authors:** Chao Chen, Qixuan Zhu, Minghui Shan, Lei Cheng, Weiwei Wang, Zhiqiang Lu, Yi Wang, Hengda Sun, Yusuke Yamauchi, Jing Tang, Guiyin Xu

**Affiliations:** 1 School of Materials Science and Engineering, Zhengzhou University, Henan Academy of Sciences, Zhengzhou, 450052 Henan People’s Republic of China; 2 State Key Laboratory of Advanced Fiber Materials, College of Materials Science and Engineering, Donghua University, Shanghai, 201620 People’s Republic of China; 3 State Key Laboratory of Petroleum Molecular and Process Engineering, Shanghai Key Laboratory of Green Chemistry and Chemical Processes, School of Chemistry and Molecular Engineering, East China Normal University, Shanghai, 200062 People’s Republic of China; 4 Australian Institute for Bioengineering and Nanotechnology (AIBN) and School of Chemical Engineering, The University of Queensland, Brisbane, QLD 4000 Australia; 5 Department of Materials Process Engineering, Graduate School of Engineering, Nagoya University, Nagoya, Japan; 6 Department of Chemical and Biomolecular Engineering, Yonsei University, 50 Yonsei-Ro, Seodaemun-Gu, Seoul, 03722 South Korea

**Keywords:** Battery recycling, Microwave plasma treatment, Heterostructure, Electrolyzed water

## Abstract

The key challenge of water electrolysis is the high energy barrier of the oxygen intermediate of the oxygen evolution reaction (OER) and the unclear catalytic active sites. The recycling of valuable metals from spent batteries and their reconstruction into high-performance electrolytic water catalysts offers significant economic and environmental benefits. In this work, we report a Co_3_O_4_/NiFe-layered double hydroxide heterostructure electrocatalyst by reconstruction of spent LiCoO_2_ cathode material through microwave plasma treatment and then compositing with NiFe-layered double hydroxides through a simple hydrothermal method. This catalyst achieves a low overpotential of 235 mV at 10 mA cm^−2^ for OER and exhibits prolonged stability at high current densities when integrated into an alkaline electrolyzer, which surpasses commercial IrO_2_. In situ spectral analysis and density functional theory demonstrate that the presence of heterostructures between Co_3_O_4_ and NiFe-LDH optimizes the charge transport path and reduces the interface resistance. Specifically, the charge redistribution optimizes the adsorption energy of oxygenated intermediates on the Ni and Fe sites, thereby optimizing the reaction pathway and significantly improving the overall OER catalytic efficiency. This work provides a green and scalable way to convert spent metal sources into high-value electrocatalysts, while addressing critical challenges in energy storage and sustainable hydrogen production.

**Supplementary Information:**

The online version contains supplementary material available at 10.1007/s40820-026-02164-1.

## Highlights

- A closed-loop recycling route for spent LiCoO_2_ batteries, converting metal resources into high-value electrocatalysts with environmental and economic merits.
- Synergistic microwave plasma etching and hydrothermal method enable precise construction of Co_3_O_4_/NiFe-LDH heterostructures, optimizing material surface and interface properties.
- Charge redistribution at the heterointerface regulates oxygen intermediate adsorption, providing a new interfacial engineering idea for low-cost water electrolysis catalysts.

**Supplementary Information:**

The online version contains supplementary material available at 10.1007/s40820-026-02164-1.

## Graphical abstract

**Figure:**
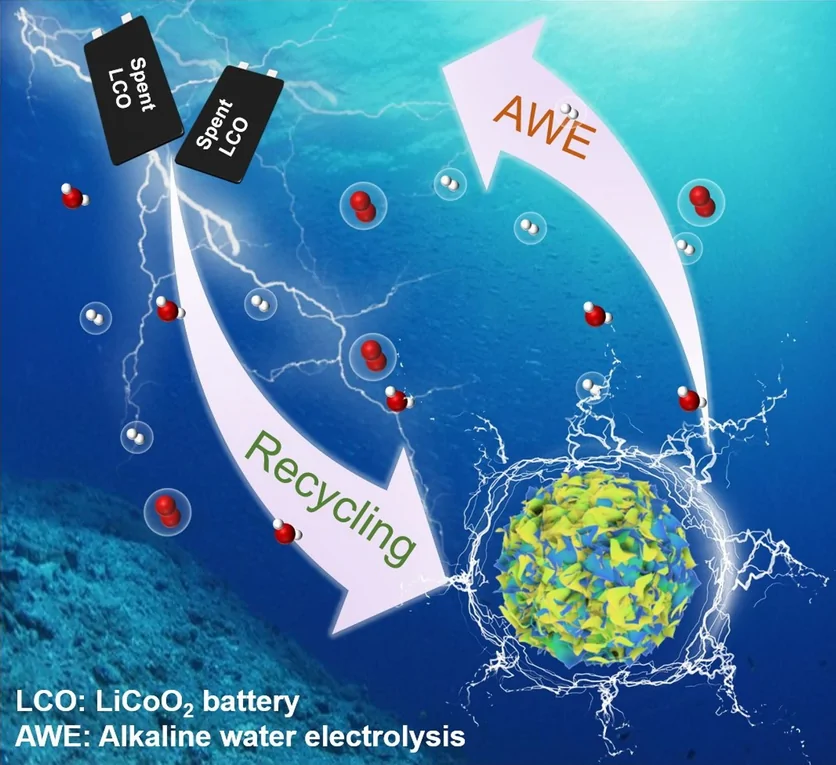

## Introduction

**Fig. 1:**
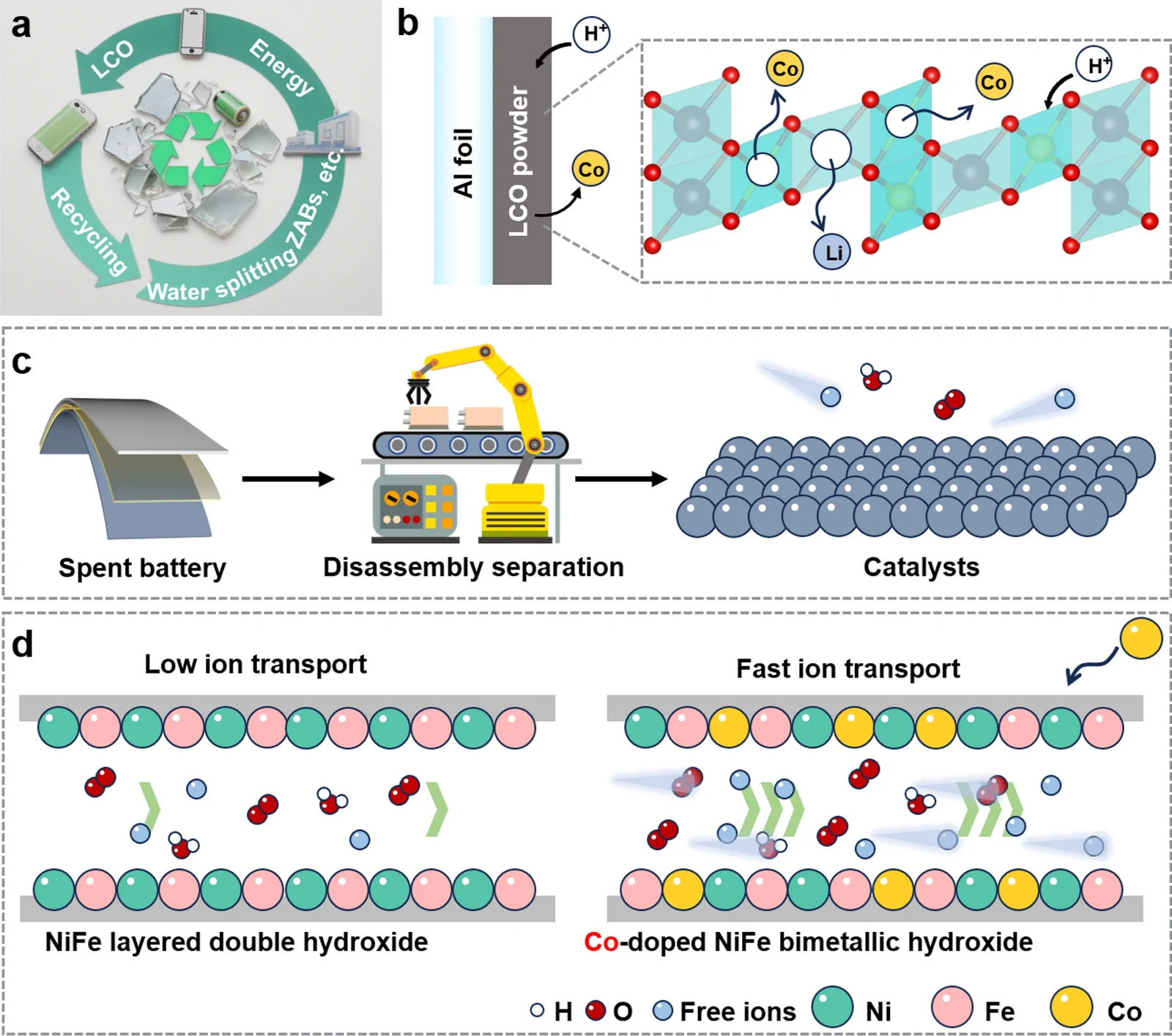
Concept of this work. **a** Significance of recycling use of spent battery cathode material. **b** Separation illustration of cobalt and lithium elements from lithium cobalt oxide. **c** Schematic diagram of preparing a catalyst by recycling spent battery cathode materials. **d** Ion transport diagram over the heterogeneous interfaces

Limited by the battery electrode material itself, the capacity of commercial lithium-ion batteries (LIBs) usually degrades to 80% of their initial capacity within 8 years, resulting in a large number of spent batteries [1–3]. The traditional landfill disposal methods not only cause the irreversible loss of strategic metal resources (including lithium, cobalt, and nickel etc.), but also pose significant environmental risks through soil contamination and groundwater pollution [4, 5]. The effective recovery of cathode materials such as lithium cobalt oxide (LiCoO_2_) [6], nickel-cobalt-manganese ternary (NCM) [7] or nickel-cobalt-aluminum oxide (NCA) [8] not only has significant economic and environmental benefits, but also conforms to the concept of closed-loop recovered resources (Fig. 1a) [9–11]. These cathode materials contain a large number of transition metal elements, which have variable oxidation states and unfilled d-electron orbitals. These characteristics promote these elements to be widely used in the field of catalysis (Fig. 1b) [12–14]. In addition, regenerated transition metals (especially cobalt-based compounds, such as Co_3_O_4_) have been proven to be transformative raw materials for the synthesis of advanced electrocatalysts, and their optimized surface morphology and catalytic activity are comparable to those of noble metal catalysts (such as IrO_2_ and Pt/C) [15–17]. This dual-effect approach not only solves the key challenges of metal resource scarcity and environmental pollution mitigation, but also creates a synergistic way to promote the development of next-generation energy technologies, especially hydrogen fuel cells, water splitting, and metal-air batteries.

At present, the recovery of cathode materials for spent lithium-ion batteries mainly relies on physical (mechanical stripping/crushing) and chemical (solvent leaching/high temperature reduction) methods. But these methods face purity bottlenecks or energy consumption/pollution problems [18–20]. It is worth noting that in recent years, the innovation of recycling technology (such as low-temperature solid water leaching [21], anion exchange membrane-assisted extraction [10], and acid leaching-sulfurization coupling [22]) has successfully converted spent cathodes into an electrocatalyst (Fig. 1c). However, when these recycled materials are used as electrolyzed water catalysts without modification, they generally have insufficient exposure of catalytically active sites and low electron transport efficiency, resulting in slow reaction kinetics and poor catalytic activity. In contrast, the ultra-high temperature (up to thousands or tens of thousands of degrees Celsius) generated by plasma technology can completely decompose complex compounds into basic elements or reduce them to simpler forms in a very short time (milliseconds), showing unique potential in recycling cathode materials of spent LIBs [23]. In addition, the construction of heterostructures (metal interface, lattice strain adjustment, etc.) has been proven to be an effective strategy to improve the performance of catalysts for alkaline electrolytic water (Fig. 1d) [24–26]. The heterogeneous interfaces have significantly improved the performance of catalytic materials through unique interface interactions. The construction of heterostructures can form strong electron coupling effects at the interface, regulating the charge distribution through the interface electric field, inducing the electron orbital reconstruction of the active site, and optimizing the adsorption/desorption energy barrier of the intermediate product (*OH, *O, *OOH, etc.) [27–30]. The lattice mismatch between different components in the metal interface can also cause lattice distortion and further enhance the intrinsic reactivity of the active sites [31]. In addition, the heterogeneous interface can form a directional charge transfer channel by adjusting the Fermi level difference, which greatly improves the electron transport efficiency [32]. This multi-scale interfacial synergistic effect not only increases the exposure of catalytically active sites, but also reduces the overpotential of hydrogen evolution/oxygen evolution reaction by optimizing the reaction path, and finally achieves efficient and stable catalytic performance of electrolytic water [33–37].

Based on the foregoing insights, a dual-engine control strategy of surface reconstruction-heterogeneous interface synergy is proposed in this study. First, Co_3_O_4_ was reconstructed from spent LIBs via microwave plasma etching, which was further coupled with NiFe-LDH nanosheets by the hydrothermal method to optimize the surface microstructure and heterogeneous interfaces. Co_3_O_4_ can be used to optimize the interface state and electronic structure of common NiFe-LDH catalysts. Through effective NiFe charge modulation, the adsorption strength of the intermediates (*OH, *O, *OOH) in the whole reaction path is synergistically adjusted to conform to the Sabatier principle, thereby improving the kinetic performance of OER. Electrochemical tests show that the optimized Co_3_O_4_/NiFe-LDH displayed an overpotential of 235 mV at 10 mA cm^−2^ in 1.0 M KOH, exceeding most reported regenerated catalysts and showing a stability of up to 24 h. In addition, the integrated anode applied in an alkaline electrolytic cell requires 1.74 V to achieve a current density of 200 mA cm^−2^ and operates stably at this current density for 140 h. This work not only proves the effectiveness of plasma-assisted engineering for material regeneration, but also provides a feasible way to promote the cost-effective development of electrolytic water hydrogen production technology through advanced interface regulation.

## Experimental Section

### Materials

Ferric nitrate (Fe(NO_3_)_3_·9H_2_O, 98%), nickel nitrate (Ni(NO_3_)_2_·6H_2_O, 98%), ammonium fluoride (NH_4_F, 98%) were purchased from Sinopharm Chemical Reagent Co., Ltd. Urea (CH_4_N_2_O, 98%), ethanol (C_2_H_6_O, 98%), iridium oxide (IrO_2_, 98%), potassium hydroxide (KOH, 95%) were purchased from Aladdin. Co., Ltd. Commercial Pt/C (20 wt%), Nafion (5 wt%) were purchased from Shanghai Macklin Biochemical Technology Co., Ltd. All chemicals used were of analytical grade and required no further purification.

### Preparation of Co_3_O_4_, NiFe-LDH and Co_3_O_4_/NiFe-LDH

#### ***Separation and Utilization of Li and Co from Batteries***

*Battery disassembly*: The spent lithium cobalt oxide soft pack battery (from iPhone 6) was immersed in 1.0 M NaCl solution for 24 h to fully discharge it. The surface packaging was slowly cut with insulated scissors to obtain the waste lithium cobalt oxide battery cathode sheet. The waste lithium cobalt oxide battery cathode sheet was immersed in a mixed solution of ethanol and deionized water (v/v = 1:1), and ultrasonicated for 30 min. The waste lithium cobalt oxide battery cathode material and the aluminum foil current collector were separated to obtain the waste lithium cobalt oxide battery cathode material, which was centrifuged and washed three times with ethanol. The spent lithium cobalt oxide battery cathode material powder (named S-LCO) was obtained by vacuum drying at 80 °C.

The theoretical recovery efficiency (*η*_Li_) can be estimated by the following formula based on material balance:

1 $${\eta }_{\mathrm{Li}}=\left(1-\frac{{m}_{\text{residual Li}}}{{m}_{\text{initial Li}}}\right)\times 100\%$$

Here, *m*_initial Li_ is the total lithium mass in the waste lithium cobalt oxide raw material, and *m*_residual Li_ is the lithium mass remaining in the solid residue after plasma treatment and water immersion. Ideally, after sufficient plasma reaction and leaching, this term should approach 0.

*Plasma:* The plasma experiment was carried out according to the previous work of the research group [23]. The microwave plasma treatment was performed in a microwave plasma system (Laboratory self-built) under an Air atmosphere (flow rate: 50 sccm) at a power of 800 W for 5 min. The temperature during the process was maintained at ~ 1300 K, monitored by an infrared pyrometer.

#### ***Synthesis of NiFe-LDH, Co***_***3***_***O***_***4***_***/NiFe-LDH Catalysts***

In order to obtain Co_3_O_4_/NiFe-LDH, 50 mg of Co_3_O_4_ powder was ultrasonically dispersed in 30 mL of deionized water, and then Ni(NO_3_)_2_ (0.5 mmol) and Fe(NO_3_)_3_ (0.5 mmol), as well as NH_4_F (2 mmol) and urea (2.5 mmol) were dissolved in the above solution. After stirring for 30 min, it was transferred to a high-pressure reactor and reacted at 120 ºC in an oven for 10 h. After the reaction, the product was collected by centrifugation, washed, and vacuum-dried at 60 ºC. Finally, Co_3_O_4_/NiFe-LDH catalyst was obtained. The synthesis process of NiFe-LDH is to hydrothermally obtain the final product without adding Co_3_O_4_ in the above steps.

### Materials Characterization

The characterization of morphology and composition of the catalysts was performed using a JEM-2100F (JEOL) microscope equipped with an energy-dispersive X-ray spectrometry (EDS) instrument at 200 kV. The crystal structure was characterized by a Bruker D8 Advance X-ray diffractometer (Cu K*α*, *λ* = 1.5406 Å) at 40 kV and 40 mA. The XRD patterns were collected in the 2*θ* range of 10–80°. X-ray photoelectron spectroscopy (XPS) was collected by Thermo VG Scientific ESCALAB 250XI spectrometer with an Al Kα radiator. All XPS signals were corrected by C 1*s* spectrum at 284.8 eV. Raman spectra were operated by Laser Microscopic Confocal Raman Spectrometer (Lab RAM HR800, *λ* = 514 nm). Inductively coupled plasma atomic emission spectrometry (ICP-OES) analysis was performed using PerkinElmer ICP 2100.

### Electrochemical Measurements

The electrochemical activity of various catalysts was investigated at room temperature using a three-electrode system with a CHI 760f electrochemical workstation (Chenhua, Shanghai). The catalyst ink was prepared by dispersing 3.5 mg catalyst in 20 μL 5 wt% Nafion solution and 0.48 mL ethanol/water (v/v = 1:1) mixed solvent under ultrasonic action for 1.5 h. The catalytic ink was gradually dropped on the surface of carbon paper (CP) with a load of about 0.98 mg cm^−2^ as the working electrode. Hg/HgO and carbon rods were used as the reference electrode and the counter electrode, respectively. All electrochemical tests were carried out in 1.0 M KOH (pH = 13.85) [38]. Linear sweep voltammetry (LSV) was performed from 0 to 1 V (vs. Hg/HgO) at a scan rate of 5 mV s^−1^. The electrochemical surface area (ECSA) was measured by CV measurement at different scan rates (10 ~ 50 mV s^−1^). Electrochemical impedance spectroscopy (EIS) measurements were performed in the range of 100 kHz to 0.1 Hz. The stability of the catalyst was evaluated by multi-potential stability test. All potentials refer to reversible hydrogen electrode (RHE), and the formula is *E* (vs. RHE) = *E* (vs. Hg/HgO) + 0.098 + 0.059 × pH.

The membrane electrode assembly was prepared by CCS (Catalyst-Coated Substrate) method for hydrogen production in alkaline electrolyzers. The specific operations are as follows: Co_3_O_4_/NiFe-LDH catalysts were used as OER electrocatalysts. Subsequently, the MEA was assembled by stacking layers in the following order: gas diffusion layer, anode catalyst layer, commercial membrane (ZIRFON UTP 500) and blank nickel foam layer, the prepared membrane electrode assembly was hot-pressed at 0.1 MPa and 70 °C. The electrochemical measurement of alkaline water electrolytic cell was carried out by using CorrTest electrochemical workstation (Wuhan CorrTest Instruments Co., Ltd.).

### Density Functional Theory Calculations

All the density functional theory (DFT) calculations were performed using CP2K code. The Perdew-Burke-Ernzerhof (PBE) exchange–correlation and DZVP basis sets combined with Goedecker-Teter-Hutter (GTH) pseudopotentials were used. The plane-wave cutoff energy was set at 500 Ry, and the self-consistent field (SCF) convergence was set to be 1.0 × 10^−6^ Ha. The DFT calculations used periodic boundary conditions in XYZ directions. The vacuum thickness for the Z direction was set as 10 Å. The D3 dispersion correction was applied to improve the van der Waals interaction. The free energy diagrams for OER were calculated with reference to the computational hydrogen electrode. The free energy of the gas phase and adsorbed species can be obtained from the following equation:

2 $$\Delta G = {\Delta E}_{\mathrm{DFT}} + \Delta \text{ZPE }-T\Delta S$$

where *E*_DFT_ was the electronic energy, T was set at 298.15 K. ∆ZPE and *T*Δ*S* were the change in the zero point energy and entropy at room temperature (T = 298.15 K), which were obtained after frequency calculations.

Charge density difference of the Co_3_O_4_/NiFe-LDH system:

3 $$\Delta \rho ={\rho }_{{\mathrm{Co}}_{3}{O}_{4}/\mathrm{NiFe}-\mathrm{LDH}} -{\rho }_{{\mathrm{Co}}_{3}{O}_{4}}-{\rho }_{\mathrm{NiFe}-\mathrm{LDH}}$$

Mechanism of OER in alkaline medium:

4 $$* + {\mathrm{OH}}^{ - } \to {\mathrm{OH}}^{*} + e^{ - } \;\Delta G_{1}$$

5 $${\mathrm{OH}}^{*} + {\mathrm{OH}}^{ - } \to O^{*} + H_{2} O \left( l \right) + e^{ - } \;\Delta G_{2}$$

6 $$O^{*} + {\mathrm{OH}}^{ - } \to {\mathrm{OOH}}^{*} + e^{ - } \;\Delta G_{3}$$

7 $${\mathrm{OOH}}^{*} + {\text{ OH}}^{ - } \to *^{ } + O_{2} \left( g \right) + H_{2} O \left( l \right)\;\Delta G_{4}$$

## Results and Discussion

### Material Synthesis and Characterizations

**Fig. 2:**
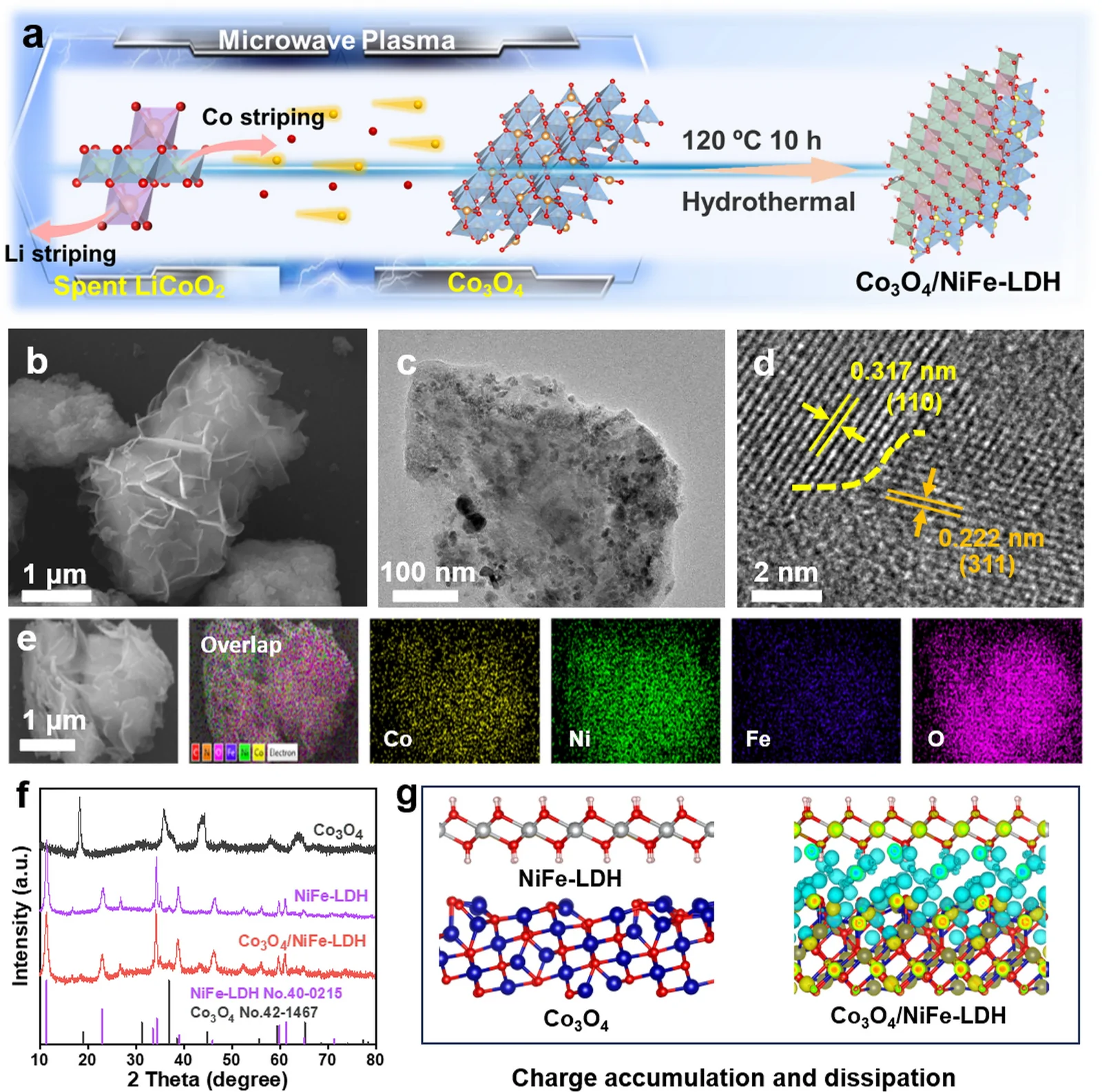
Synthesis process and characterization of microstructures. **a** Plasma treatment of LiCoO_2_ cathode material and the sketch map of Co_3_O_4_/NiFe-LDH catalyst synthesis. **b** Scanning electron microscope image of Co_3_O_4_/NiFe-LDH. **c, d** High-resolution TEM image of Co_3_O_4_/NiFe-LDH. **e** Corresponding elemental mapping of Co, Ni, Fe and O for Co_3_O_4_/NiFe-LDH. **f** XRD patterns of the Co_3_O_4_, NiFe-LDH and Co_3_O_4_/NiFe-LDH. **g** Optimized crystal structure models and charge density difference analysis of Co_3_O_4_/NiFe-LDH, Yellow and cyan isosurfaces represent regions of electron accumulation and depletion. The blue, gray and yellow spheres represent Co, Ni, and Fe atoms, respectively (See Fig. S21 for the corresponding line-scan profile and quantitative analysis)

The synthesis process of Co_3_O_4_/NiFe-LDH heterostructure includes the plasma recovery of spent lithium cobalt oxide batteries and further coupling with NiFe-LDH nanosheets via hydrothermal (Fig. 2a). In this process, the dismantled spent lithium cobalt oxide batteries are placed in ethanol/water solution (v/v = 1:1), and the leaching begins at 80 °C, selectively recovering lithium ions and leaving cobalt-rich solid residues. In this process, lithium ions are preferentially extracted (forming lithium complexes), leaving cobalt-rich solid residues (mainly containing Co(III) oxide precursor). The ethanol-water mixed solvent can significantly reduce the dielectric constant of the system and weaken the solvation ability of water molecules to Co^3+^. Li preferentially forms [Li(H_2_O)_*x*_(EtOH)_*y*_]^+^ complex due to the low solvation energy (Li leaching rate ˃ 90%) [39]. The residual cobalt species were subsequently converted into porous Co_3_O_4_ through microwave plasma treatment, which is confirmed by XRD analysis [40]. The high-energy plasma environment provides rapid localized heating and ion bombardment, which promotes the decomposition of Co(III)-rich residues into Co_3_O_4_. This process avoids high-temperature sintering and preserves a high surface area. Finally, vertically aligned NiFe-LDH nanosheets were epitaxially grown on the Co_3_O_4_ substrate via hydrothermal reaction using nickel nitrate and iron nitrate as precursors, forming a heterostructure through synergistic interfacial interactions. For comparison, NiFe-LDH was synthesized without adding porous Co_3_O_4_ in the hydrothermal process.

The morphologies of the products from different synthetic stages were observed by scanning electron microscopy (SEM). The Co_3_O_4_ obtained after microwave plasma treatment exhibits a rough surface block structure (mean particle size ~ 1 μm, Fig. S1a). NiFe-LDH fabricated via the hydrothermal method shows wrinkled microspheres with a diameter of about 2 μm (Fig. S1b). SEM imaging reveals that the vertically arranged NiFe-LDH nanosheets grown uniformly on the Co_3_O_4_ substrate and formed a three-dimensional porous network (Fig. 2b). The specific exposed crystal plane of the Co_3_O_4_ substrate is matched with the lattice of LDH, which induces the LDH layer to grow nearly perpendicular to the substrate surface [41]. In this process, chemical bonding and strong electrostatic interaction will form between the NiFe-LDH laminate and the surface atoms of the Co_3_O_4_ substrate. This interfacial interaction is not only the key to stabilizing the heterostructure, but also may significantly affect the electronic structure of the composite material and optimize its electrocatalytic performance [42].

Transmission electron microscopy (TEM) further confirms the ultrathin properties of the NiFe-LDH nanosheets (thickness < 5 nm, Fig. S2) and their close interface contact with Co_3_O_4_, which is consistent with the designed heterostructure (Fig. 2c). High-resolution TEM (HRTEM) images of Co_3_O_4_/NiFe-LDH composite reveal that the lattice fringes are spaced at 0.222 and 0.317 nm, corresponding to the (311) crystal plane of Co_3_O_4_ and the (110) crystal plane of NiFe-LDH, respectively (Fig. 2d) which is further confirmed by the additional structural analysis provided in Figs. S3 and S4. The apparent boundary region between NiFe-LDH and Co_3_O_4_ in the image confirms the successful formation of heterostructure, which will enhance electron transfer and generate new catalytically active sites at the heterogeneous interface of OER [43]. In addition, there is a significant difference between the interplanar spacing of Co_3_O_4_ (311) and NiFe-LDH (110) (~ 30% lattice mismatch), but this mismatch introduces a controllable lattice strain at the interface. The strain and the chemical bonding and electrostatic interaction between the interfaces jointly induce significant electron reconstruction, thereby optimizing the electrocatalytic performance of the composites. Energy-dispersive X-ray spectroscopy (EDS) mapping of the Co_3_O_4_/NiFe-LDH composite (Fig. 2e) demonstrates the homogeneous distribution of Co, Ni, Fe, and O elements throughout the sample. Furthermore, inductively coupled plasma optical emission spectrometry (ICP-OES) analysis determined the content of various elements, revealing Co/Ni/Fe molar ratio of 1: 10.58: 3.37 in the final product (Table S1).

As shown in Fig. 2f, the phase structure and microstructure of Co_3_O_4_, NiFe-LDH, and Co_3_O_4_/NiFe-LDH were characterized by X-ray diffraction (XRD). The XRD pattern of Co_3_O_4_ exhibited sharp and intense diffraction peaks, indicating high crystallinity and large grain size (JCPDS card No. 42-1467) [44]. In contrast, NiFe-LDH shows the characteristic peaks with low intensity, indicating that it has low crystallinity (JCPDS card No. 40-0215), and TEM images verifies that it mainly exists in the form of nanosheets [45]. The diffraction peak at 26.5° may be a new long-range ordered structure induced by the strong interface coupling between Co_3_O_4_ and NiFe-LDH. The XRD pattern of the Co_3_O_4_/NiFe-LDH reveals diffraction peaks positioned between those of the individual components, and these peaks correspond to a combination of the characteristic peaks from both Co_3_O_4_ and NiFe-LDH, which confirms the existence of the heterostructure. Notably, within the Co_3_O_4_/NiFe-LDH composites, the characteristic peak strength of Co_3_O_4_ decreases and the peak width increases, possibly due to lattice distortion caused by interfacial interactions (such as the occurrence of cation doping) [46]. Furthermore, Fig. 2g depicts the atomic arrangement and electron redistribution at the Co_3_O_4_/NiFe-LDH heterogeneous interface, showing the optimized crystal structure model (upper panel) and the charge density difference distribution (lower panel). Isosurfaces in yellow (indicating electron accumulation) and cyan (indicating electron depletion) clearly visualize the aggregation and depletion of electrons at the interface. The image reveals the spatial path of electron transfer originating from the Fe sites (cyan, depletion) through the interface to the Ni sites (yellow, accumulation), consistent with the Fe → Ni electron transfer direction inferred from XPS analysis. This observed charge redistribution indicates strong electronic coupling at the Co_3_O_4_/NiFe-LDH interface, likely responsible for its superior performance compared to individual Co_3_O_4_ and NiFe-LDH components. Similarly, the charge redistribution near Fe and Ni atoms in Co_3_O_4_/NiFe-LDH indicates that the incorporation of Co_3_O_4_ changes the electronic structure, which provides strong proof for the subsequent improvement of OER activity. This analysis clearly reveals the spatial pattern of electron gain and loss around the constituent atoms in the interface region, and provides atomic-scale visual evidence for the interfacial electron transfer.

**Fig. 3:**
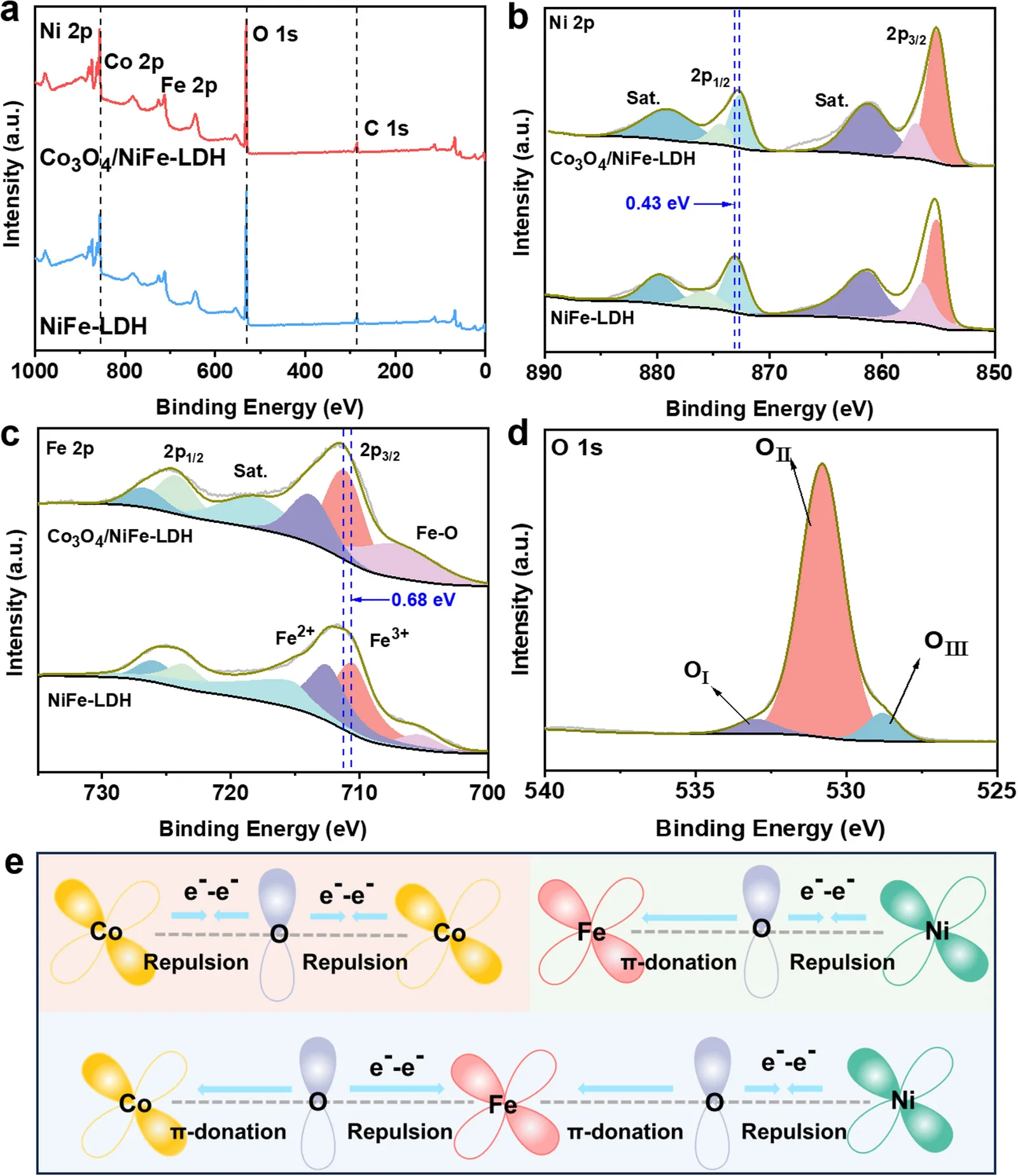
Structural characterizations of catalysts. **a** XPS patterns of the NiFe-LDH and Co_3_O_4_/NiFe-LDH. XPS spectra of **b** Ni 2*p*, **c** Fe 3*d*, and **d** O 1*s*. **e** The electronic coupling diagram of Co_3_O_4_, NiFe-LDH, and Co_3_O_4_/NiFe-LDH

XPS was used to further study the chemical state and coordination environment of the catalyst, revealing the trend of the OER pathway. The presence of Co, Ni, Fe, and O elements in the measurement spectrum of the sample (Fig. 3a), corroborated by EDS results, strongly confirms the successful synthesis of the composite material. In the high-resolution Co 2*p* spectrum (Fig. S5), the characteristic doublet peaks at 780.6 eV (Co 2*p*_3/2_) and 785.3 eV (Co 2*p*_1/2_) correspond to Co^3+^ and Co^2+^ species in Co_3_O_4_. The minor peak observed at 774.8 eV suggests the potential formation of metallic Co, possibly arising from partial reduction during synthesis[47, 48]. The high-resolution Ni 2*p* spectrum of Co_3_O_4_/NiFe-LDH exhibited two fitting peaks of 855.21 and 872.47 eV are attributed to Ni 2*p*_3/2_ and Ni 2*p*_1/2_, respectively (Fig. 3b). It is worth noting that compared with the original NiFe-LDH, the intensity of the Ni characteristic peak of Co_3_O_4_/NiFe-LDH is reduced and shifted (0.43 eV), which may be due to the electrostatic attraction or coordination bond formation at the interface with Co^3+^, resulting in a change in the electron cloud density around Ni [45]. In the high-resolution Fe 2*p* spectrum of Co_3_O_4_/NiFe-LDH (Fig. 3c), the two fitting peaks of 711.26 and 724.47 eV are attributed to Fe 2*p*_3/2_ and Fe 2*p*_1/2_, respectively. The peaks of Fe 2*p* move significantly in the direction of high binding energy (0.68 eV), indicating that the electron cloud density around Fe decreases in Co_3_O_4_/NiFe-LDH compared with NiFe-LDH. These shifts indicate that the strong electronic coupling between Co_3_O_4_ and NiFe-LDH leads to the redistribution of charge at the interface, which optimizes the electronic structure of the active sites of Ni and Fe rather than causing a complete change in their oxidation states [35]. Finally, the O 1*s* high-resolution spectrum displayed three distinct peaks at 533.1, 530.8, and 528.8 eV, these are assigned adsorbed oxygen (O_Ⅰ_) that water molecules and hydroxyl groups adsorbed on the catalysts surface, defect oxygen (O_ⅠⅠ_), and Lattice-oxygen (O_ⅠⅠⅠ_) (Fig. 3d) [15, 49, 50]. In order to elucidate the specific electron transfer mechanism, we established a Co–O–Fe–O–Ni unit model to analyze the electron interaction at the interface (Fig. 3e). At the interface, there is a strong electron repulsion between the *d*_xz_ orbitals of Fe^3+^ (3*d*^5^, pink marker) and Co^3+^ (3*d*^6^, yellow marker), resulting in a decrease in the electron cloud density at the Fe site. Concurrently, the bridging O^2−^ (purple marker) donates its *π* electrons via the 2*p* orbital to the vacant dx^2^−y^2^ orbitals of the adjacent Co^3+^. This synergy of orbital repulsion and electron donation drives electron migration along the pathway Fe → O → Co → Ni, ultimately forming an electron-enriched region at the Ni^2+^ site. This process optimizes the d-band center position of the metal active center by reconstructing the interface charge distribution and significantly reduces the adsorption energy barrier of the oxygen intermediate, thereby improving the intrinsic activity of the oxygen evolution reaction.

### Electrochemical Characterization in Alkaline Electrolyte

**Fig. 4:**
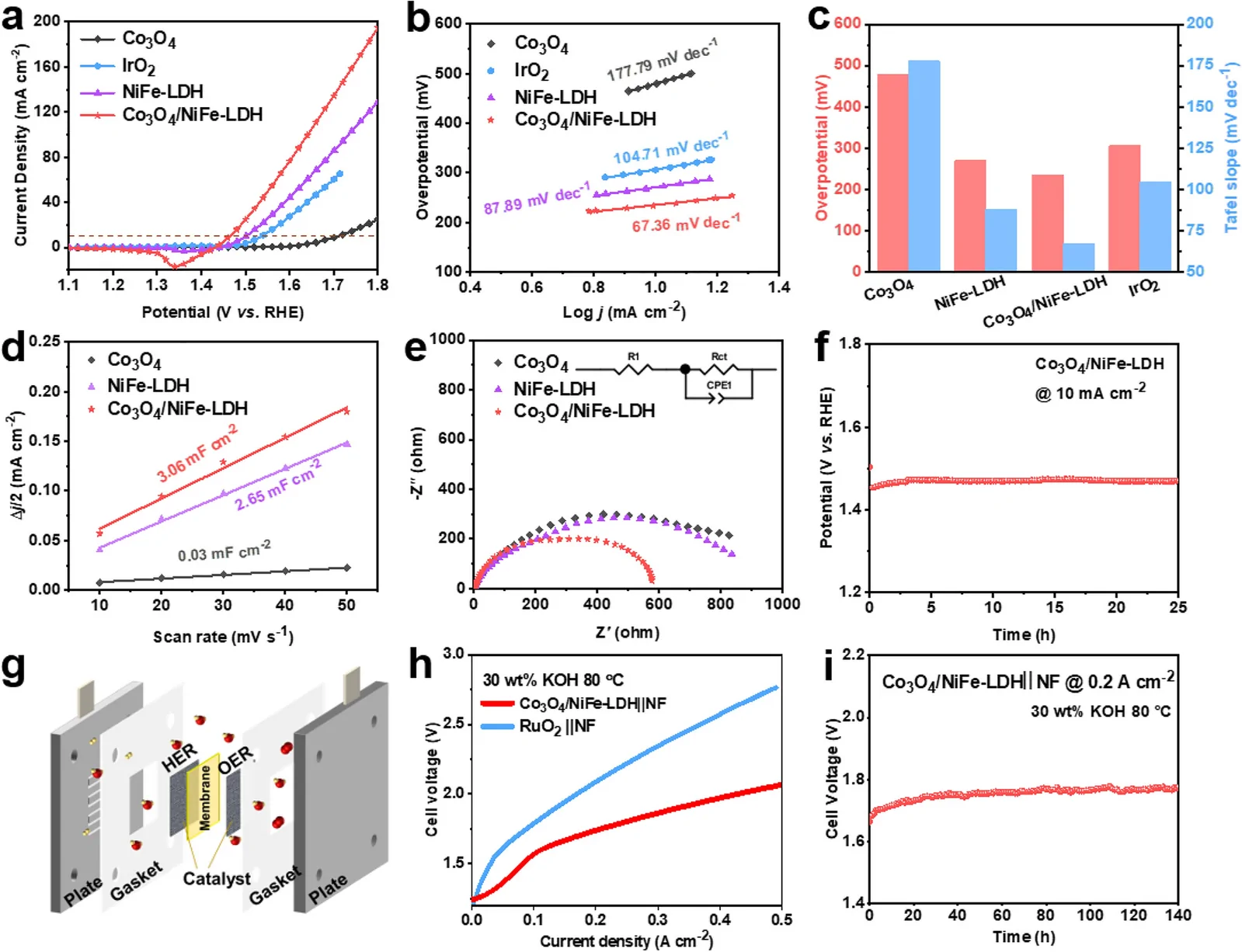
OER performance of Co_3_O_4_, NiFe-LDH, Co_3_O_4_/NiFe-LDH, and IrO_2_ catalysts. **a** OER polarization curves. **b** corresponding Tafel plots. **c** Comparison of the overpotentials and Tafel plots at 10 mA cm^−2^. **d** Double-layer capacitance (*C*_dl_) for ECSA. **e** Nyquist plots with the fitting curves, and **f** stability test of the Co_3_O_4_/NiFe-LDH for OER at 10 mA cm^−2^ in 1.0 M KOH. Electrolysis performance of the MEAs.** g** Schematic diagram of alkaline water electrolysis. **h** Polarization curves of Co_3_O_4_/NiFe-LDH||NF and RuO_2_||NF in 30 wt% KOH at 80 ºC. **i** Stability test of Co_3_O_4_/NiFe-LDH in 30 wt% KOH at 200 mA cm.^−2^

To evaluate the alkaline OER properties of the synthesized Co_3_O_4_/NiFe-LDH catalyst, a series of tests and characterization were performed in 1.0 M KOH electrolyte (The final catalyst photograph is shown in Fig. S6). For comparison, commercial IrO_2_ catalysts attached to carbon paper and other comparison samples were also studied. We first tested its redox properties by cyclic voltammetry (Fig. S7). Subsequently, the OER polarization curves of different catalysts are shown in Fig. 4a. The Co_3_O_4_/NiFe-LDH catalyst exhibits superior performance, requiring only 235 mV (vs. RHE) to achieve a current density of 10 mA cm^−2^. This significantly outperforms the NiFe-LDH catalyst (270 mV), the Co_3_O_4_ catalyst (479 mV), and even the benchmark IrO_2_ catalyst (306 mV). The comparison of Tafel slopes and overpotentials for each sample is shown in Fig. 4b, c. The Co_3_O_4_/NiFe-LDH catalyst consistently demonstrates excellent catalytic activity. This enhancement indicates that the synergistic electronic interaction between Co_3_O_4_ and NiFe-LDH may be due to optimized charge transfer at the heterogeneous interface rather than simple physical mixing (Fig. S8). The Tafel slope value further supports that the composite follows the conventional adsorbate evolution mechanism (AEM), where the reaction kinetics are governed by sequential surface intermediate formation (*OH → *O → *OOH), consistent with the observed kinetic barrier reduction [51]. In addition, Co_3_O_4_/NiFe-LDH also performed better than most other reported literatures (Table S3). In addition, the electrochemically-active surface area (ECSA) was estimated by cyclic voltammetric (CV)-evaluated double-layer capacitance (*C*_dl_) to further elucidate the high OER performance of Co_3_O_4_/NiFe-LDH (Fig. S9). As shown in Fig. 4d, the *C*_dl_ value of Co_3_O_4_/NiFe-LDH is 3.06 mF cm^−2^, which is higher than that of NiFe-LDH (2.65 mF cm^−2^) and Co_3_O_4_ (0.03 mF cm^−2^), indicating that there are more active sites of Co_3_O_4_/NiFe-LDH. These results suggest that the synergy of NiFe-LDH and Co_3_O_4_ promotes the increase in reactive sites. In order to clarify whether the performance improvement is due to the increase in the number of active sites or the enhancement of intrinsic activity, we normalized the current density of the LSV curve with the electric double layer capacitance to compare its intrinsic activity (Fig. S10). The ECSA-normalized current density of Co_3_O_4_/NiFe-LDH is significantly higher than that of NiFe-LDH. This indicates that the excellent OER performance of the composite material not only comes from the larger electrochemical active area, but also the intrinsic catalytic activity of each active site has been effectively improved.

To further understand the reaction kinetics in the OER process, electrochemical impedance spectroscopy (EIS) tests were performed to probe the interface properties and determine the electron transport process. The Nyquist plot results obtained when the overpotential is 300 mV are shown in Fig. 4e. The Co_3_O_4_/NiFe-LDH catalyst shows a smaller semicircle, indicating that the interface barrier to mass transfer and charge transfer kinetics are the lowest. Moreover, it has a smaller charge transfer resistance (*R*_ct_), which has a faster electron transfer rate to promote the OER process at the reaction interface. The long-term OER stability of Co_3_O_4_/NiFe-LDH electrode was investigated by chronopotentiometry at a current density of 10 mA cm^−2^ in alkaline electrolyte for 24 h. As shown in Fig. 4f, the overpotential remains stable throughout the test period, indicating that Co_3_O_4_/NiFe-LDH has excellent durability during OER process. In addition, the test electrode did not show any obvious structural changes, SEM and EDS showed that the three-dimensional structure was intact, Ni, Fe, Co, O and other elements were evenly distributed, and had good stability (Figs. S11 and S12). The FTIR results show that the main crystal skeleton of the composite material maintains good integrity after the electrochemical cycle, and no serious phase transition or structural collapse occurs (Fig. S13). The stability test results of the contrast sample IrO_2_ are shown in Fig. S14, and the voltage has a significant increase within 20 h. The stability of this material is mainly due to its unique layered structure: Ni-Fe bimetallic cations build a stable three-dimensional network framework by forming strong coordination bonds with interlayer oxygen vacancies, thus significantly improving the overall structural integrity of the material system [52, 53].

Furthermore, to assess the practical application potential of the Co_3_O_4_/NiFe-LDH catalyst in water electrolysis, membrane electrode assemblies (MEAs) were fabricated using the catalyst-coated substrate (CCS) method (Fig. S15). Here, Co_3_O_4_/NiFe-LDH was used as the anode catalyst layer. The MEA was assembled by stacking layers in the following order: gas diffusion layer, anode catalyst layer, commercial membrane (ZIRFON UTP 500), and blank nickel foam layer. The prepared membrane electrode assembly was hot-pressed at 0.1 MPa and 70 °C. The film-forming electrode assembly was assembled for alkaline water electrolysis to study its practical application performance (Fig. 4g). To simulate industrial operating conditions, the electrolyzer system is equipped with a temperature control system to ensure that the temperature is maintained at 80 °C, and the circulation system drives 30 wt% KOH electrolyte. The polarization curve shows that Co_3_O_4_/NiFe-LDH||NF exhibits good performance at 80 °C, reaching 1.74 V at 200 mA cm^−2^, which is better than the contrast RuO_2_||NF (Fig. 4h). In addition, the stability of hydrogen production from electrolytic water is crucial for practical applications. The Co_3_O_4_/NiFe-LDH||NF alkaline water electrolyzer exhibits excellent stability at a current density of 200 mA cm^−2^, and the voltage attenuation rate within 140 h is only 1.5 mV h^−1^ (Fig. 4i), which has certain practicality. This work confirms that the prepared Co_3_O_4_/NiFe-LDH heterojunction catalyst has excellent cycle stability over 140 h at an industrial-related current density of 200 mA cm^−2^. The post-test characterization of the system (XRD, XPS) shows that its structure and heterogeneous interface remain intact during long-term operation, which is the structural basis of its excellent durability (Figs. S16 and S17). The preliminary test at 500 mA cm^−2^ also shows a stable potential output (Fig. S18). In future, the accelerated aging research under > 1 A cm^−2^ in the optimized high current test device will be a key step to promote the large-scale application of the catalyst.

### Mechanism Analysis

**Fig. 5:**
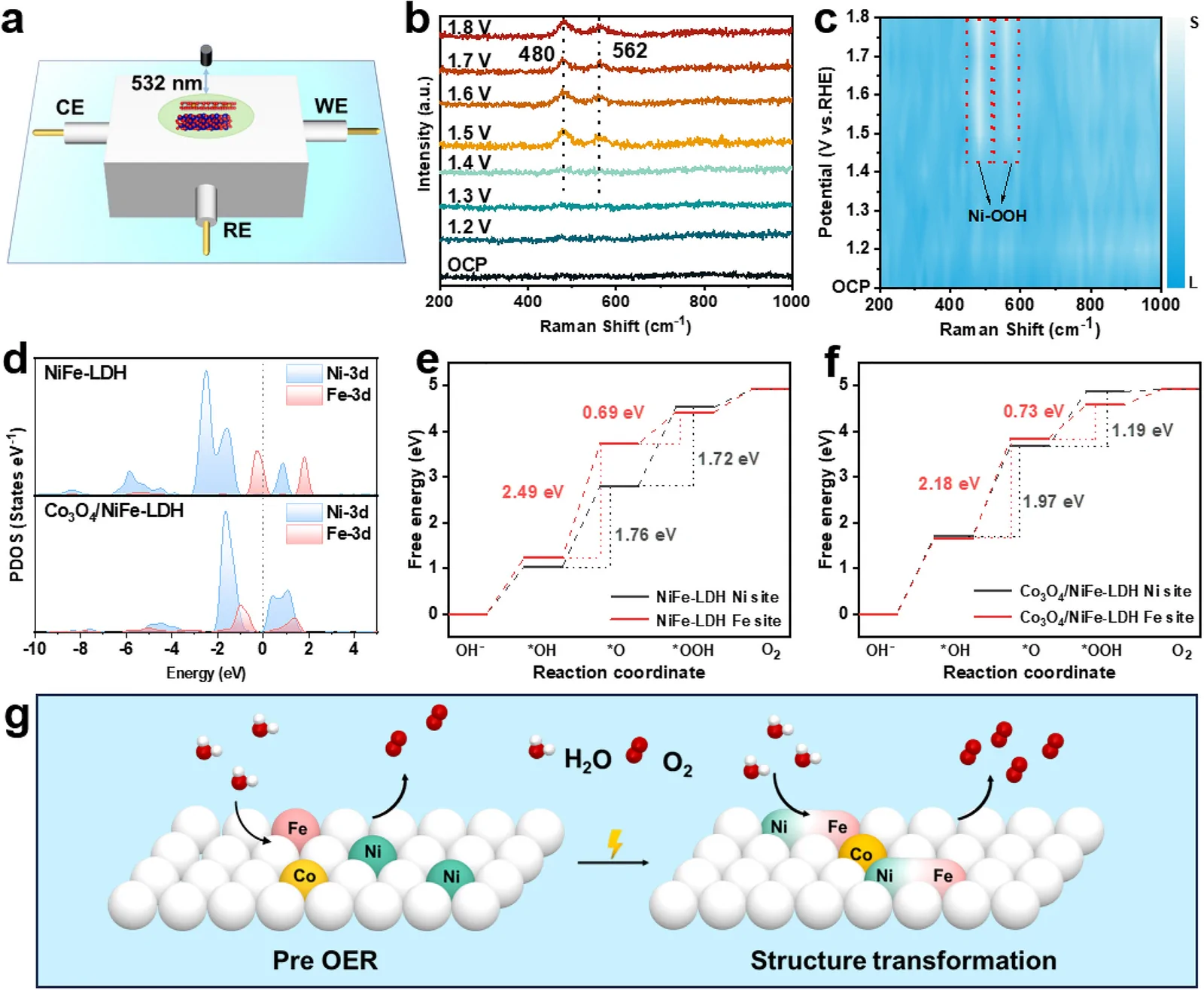
In situ characterization and theoretical calculation principle analysis. **a** In situ Raman test schematic diagram. **b** In situ Raman Spectrum measurements of Co_3_O_4_/NiFe-LDH for OER in 1.0 M KOH. **c** In situ Raman intensity maps of Co_3_O_4_/NiFe-LDH at different voltages. **d** PDOS of NiFe-LDH and Co_3_O_4_/NiFe-LDH. **e** OER pathways and energy barriers of NiFe-LDH. **f** OER pathways and energy barriers of Co_3_O_4_/NiFe-LDH. **g** Catalytic mechanism of NiFe-LDH and Co_3_O_4_/NiFe-LDH

To further elucidate the mechanism of surface dynamic reconstruction, in situ Raman spectroscopy and corresponding mapping of the catalyst were performed at an open circuit potential (OCP) and an applied potential (1.2–1.8 vs. RHE) of 0.1 V increments. More detailed information about the local environment of the Co_3_O_4_/NiFe-LDH catalyst was obtained by using in situ Raman spectroscopy, revealing the real active sites of the catalyst (Fig. 5a). The significant Raman peaks at 480 and 562 cm^−1^ correspond to the *E*_*g*_ bending vibration mode of Ni(Fe)-O, indicating that the catalytically active high-valence Ni substance is formed at the anode potential (NiFeOOH). Potential-dependent spectral evolution: At the open circuit potential (OCP), the weak Co_3_O_4_ and Ni-OOH signals indicate that it is mainly a layered double hydroxide (LDH) phase. Upon increasing the potential to 1.4–1.6 V (*vs.* RHE), the intensity of the Ni-OOH peak (562 cm^−1^) increases significantly, which is related to the electrochemical activation of NiFe-LDH into Ni-OOH under OER conditions. This shift signifies the transition from NiFe-LDH to NiFeOOH (Fig. 5b) [54, 55]. A contour map representation of the potential-dependent spectral evolution (Fig. 5c) provides further insight. It reveals a clear intensity gradient for the key Ni-OOH peak (562 cm^−1^) within the potential window of 1.6 to 1.8 V. Notably, the strengthening region in this contour map (demarcated by dotted lines) correlates with the pronounced Ni-OOH signal increase and aligns with the accelerated surface reconstruction dynamics occurring at higher potentials. In situ Raman confirmed that Co_3_O_4_/NiFe-LDH was reconstructed into Ni(Fe)OOH active phase under OER conditions. The introduction of Co_3_O_4_ not only reduces the reconstruction energy barrier by promoting the interface charge transfer, but also pre-modulates the d-band structure of the Ni/Fe site at the electronic level. This modulation effect is inherited by the reconstructed surface phase, thereby optimizing the adsorption energy of the oxygen intermediate and improving the intrinsic activity. These findings support the formation of a reconstructed Co_3_O_4_/NiFeOOH structure, which is the reason for enhanced OER performance and long-term stability.

We used DFT calculations to analyze Co_3_O_4_/NiFe-LDH composites to elucidate the relationship between electronic structure and enhanced alkaline OER activity, so as to better understand how the synergistic effect of heterogeneous interfaces promotes the improvement of OER activity observed in the material. In addition, the focus of the DFT model in this study is to reveal the regulation of the two-site reaction pathway of Ni/Fe at the heterojunction interface. Figures S19 and S20 show the optimized structures of each catalyst during the OER process. To establish the relationship between the electronic structure and OER performance, the projected density of states (PDOS) is analyzed. PDOS calculated by DFT (Fig. 5d) shows that the density of states of Fe 3*d* orbitals near the Fermi level is significantly lower than that of NiFe-LDH in Co_3_O_4_/NiFe-LDH. The decrease in the density of states of Fe 3*d* indicates that the d electron charge delocalization is enhanced, which leads to the weakening of the adsorption of oxygen-containing intermediates at the Fe site, thereby optimizing the reaction energy barrier. Further analysis shows that there is a significant hybridization between the Ni 3*d* orbital and the Fe 3*d* orbital in the energy range of −2–0 eV. The synergistic effect between the metals promotes charge transfer, thereby significantly improving the catalytic activity.

The free energy diagrams of Ni and Fe sites on Co_3_O_4_/NiFe-LDH and NiFe-LDH models were calculated (Fig. 5e, f). The diagram shows that the conversion of Ni site from *OH → *O needs to overcome a higher energy barrier (1.76 eV) in NiFe-LDH, resulting in a sharp increase in the free energy of the entire OER path, indicating that this step is the OER reaction rate-determining step (RDS). While the combined experimental and theoretical analyses support an AEM-dominated pathway, possible contributions from lattice oxygen participation cannot be completely ruled out under anodic conditions. Ni needs to overcome the high-energy barrier deprotonation behavior during the oxidation process, and the binding of the *O intermediate to the Ni site is too strong, resulting in difficulty in desorption; at the same time, the Fe site also has a high energy barrier (2.49 eV) in this step, which has a weak contribution to the overall OER activity. In contrast, the combination of Co_3_O_4_ and NiFe-LDH increases the *OH → *O energy barrier of the Ni site to 1.97 eV, and the Ni site energy barrier in the *O → *OOH step is significantly reduced, optimizing the electronic structure of O and promoting the formation of O–O bonds. At the same time, the energy barrier of the Fe site (2.18 eV) is significantly lower than that of NiFe-LDH, which reduces the desorption energy barrier. The interfacial Co_3_O_4_ induces lattice strain and electron redistribution, driving Ni–Fe hybridization and barrier optimization. This synergistic interface regulation optimizes the *O → *OOH step reaction kinetics of the Ni site, promotes the formation of O–O bonds, and reduces the RDS energy barrier of the Fe site, which reduces the energy barrier difference of the NiFe double site, promotes the parallel reaction of the dual active center, and breaks through the single RDS limit, improving OER activity (Fig. 5g).

## Conclusions

In summary, the collaborative technology system of electrode material resource regeneration and high value-added catalyst preparation for spent lithium cobaltate battery was successfully established in this study by using plasma activation technology and hydrothermal assembly process. Specifically, the efficient leaching of metal and in situ reconstruction of cobalt species in electrode materials were achieved by using the mixed solution of alcohol and water and the etching effect of microwave plasma. By accurately adjusting the hydrothermal reaction kinetic parameters, NiFe-LDH nanosheet arrays were vertically grown on the surface of defective Co_3_O_4_ to form a three-dimensional heterogeneous interface structure. Experimental characterization confirmed that the catalyst formed a continuous electron transfer channel through interfacial interaction, and its unique heterogeneous interface engineering resulted in a significant electronic synergistic effect between the NiFe-LDH sheet and the Co_3_O_4_ nanocrystals. The test of oxygen evolution reaction in alkaline medium shows that the optimized sample only needs 235 mV overpotential at 10 mA cm^−2^ current density, and can run stably for 24 h without obvious activity attenuation. It also has practical application value in alkaline electrolyzers. This performance advantage is due to the dynamic coordination reconstruction of the bimetallic active site and the rapid mass transfer at the heterogeneous interface. This study provides a new paradigm for the high-value utilization of spent lithium battery resources. This strategy has potential applicability to other cathode chemistry. For NCM, the multi-metal system may directly form high-entropy oxides after plasma treatment. For LFP, phosphorus may volatilize under the action of plasma, leaving iron oxides that can be coupled with NiCo-LDH. From simple material regeneration to precise design of high-value functional materials, it is of great practical significance to promote closed-loop circular economy and green hydrogen production technology driven by renewable energy.

## Supplementary Information

Below is the link to the electronic supplementary material.

Supplementary file1 (DOCX 39366 kb)

## References

[1] K.S. Bejigo, K. Bhunia, J. Kim, C. Lee, S. Back et al., Upcycling end of lithium cobalt oxide batteries to electrocatalyst for oxygen reduction reaction in direct methanol fuel cell *via* sustainable approach. J. Energy Chem. **82**, 148–157 (2023). 10.1016/j.jechem.2023.03.042

[2] M. Huang, M. Wang, L. Yang, Z. Wang, H. Yu et al., Direct regeneration of spent lithium-ion battery cathodes: from theoretical study to production practice. Nano-Micro Lett. **16**(1), 207 (2024). 10.1007/s40820-024-01434-0 10.1007/s40820-024-01434-0 PMC11143129 38819753

[3] Y. Cao, J. Li, H. Ji, X. Wei, G. Zhou et al., A review of direct recycling methods for spent lithium-ion batteries. Energy Storage Mater. **70**, 103475 (2024). 10.1016/j.ensm.2024.103475

[4] J.J. Roy, S. Rarotra, V. Krikstolaityte, K.W. Zhuoran, Y.D. Cindy et al., Green recycling methods to treat lithium-ion batteries E-waste: a circular approach to sustainability. Adv. Mater. **34**(25), 2103346 (2022). 10.1002/adma.202103346 10.1002/adma.202103346 34632652

[5] Y. Zhang, J. Li, W. Zhao, T. Yan, L. Zhang et al., Complete metal recycling from lithium-ion batteries enabled by hydrogen evolution catalyst reconstruction. J. Am. Chem. Soc. **145**(50), 27740–27747 (2023). 10.1021/jacs.3c10188 38059924 10.1021/jacs.3c10188

[6] J. Wang, K. Jia, J. Ma, Z. Liang, Z. Zhuang et al., Sustainable upcycling of spent LiCoO_2_ to an ultra-stable battery cathode at high voltage. Nat. Sustain. **6**(7), 797–805 (2023). 10.1038/s41893-023-01094-9

[7] Y. Xu, Y. Tian, S. Guo, B. Xu, Y. He et al., Recycling of valuable metals from spent ternary Li-ion batteries for the multi-active site electrocatalysts with high-entropy coordination. Appl. Catal. B Environ. Energy **365**, 124976 (2025). 10.1016/j.apcatb.2024.124976

[8] J. Zhu, Y. Wang, Y. Huang, R.B. Gopaluni, Y. Cao et al., Data-driven capacity estimation of commercial lithium-ion batteries from voltage relaxation. Nat. Commun. **13**(1), 2261 (2022). 10.1038/s41467-022-29837-w 35477711 10.1038/s41467-022-29837-w PMC9046220

[9] Z. Wang, Y. Chen, F. Zhou, R. Qin, Y. Tian et al., Upcycling spent lithium-ion battery cathodes into cobalt-polyphenol networks by DES dissolution and solvent-induced crystallization. Green Chem. **26**(10), 5988–5996 (2024). 10.1039/d4gc01036a

[10] L. Zhang, Q. Xu, S. Wen, H. Zhang, L. Chen et al., Recycling spent ternary cathodes to oxygen evolution catalysts for pure water anion-exchange membrane electrolysis. ACS Nano **18**(33), 22454–22464 (2024). 10.1021/acsnano.4c07340 39129247 10.1021/acsnano.4c07340

[11] K. Shen, T. Zhang, Y. Zhang, W. Tang, W. Dai et al., A sustainable net-negative carbon recycling strategy for spent batteries enabled by paired electrolysis. Chem. Eng. J. **516**, 164000 (2025). 10.1016/j.cej.2025.164000

[12] T. Zhang, K. Yang, C. Wang, S. Li, Q. Zhang et al., Nanometric Ni_5_P_4_ clusters nested on NiCo_2_O_4_ for efficient hydrogen production via alkaline water electrolysis. Adv. Energy Mater. **8**(29), 1801690 (2018). 10.1002/aenm.201801690

[13] C. Wang, M. Qiu, G. Liang, H. Yan, J. Ma et al., Interfacial electron modulation with ionic liquids: dual optimization of CO_2_ confinement and charge transfer for enhanced electroreduction on Cu. Langmuir **41**(34), 22874–22885 (2025). 10.1021/acs.langmuir.5c02334 40825308 10.1021/acs.langmuir.5c02334

[14] G. Ding, Z. Wang, Z. Chen, Y. Xiao, X. Liu et al., Unsaturated coordination-regulated high-spin nickel sites for selective solar-driven carbon dioxide conversion in pure water. Energy Environ. Sci. **19**(2), 486–495 (2026). 10.1039/d5ee04331j

[15] W. Zhao, Q. Zhang, Y. Zhu, P. Zhao, B. Chen et al., Boosting reaction kinetics and mass transfer of bifunctional co-based oxygen electrocatalyst prepared from CoAl-LDH. Adv. Energy Mater. **13**(35), 2301580 (2023). 10.1002/aenm.202301580

[16] J. Huang, A.H. Clark, N. Hales, C.N. Borca, T. Huthwelker et al., Spectroscopic investigations of complex electronic interactions by elemental doping and material compositing of cobalt oxide for enhanced oxygen evolution reaction activity. Adv. Funct. Mater. **34**(44), 2405384 (2024). 10.1002/adfm.202405384

[17] R. Lv, L. Wang, J. Lan, Z. Zhao, X. Liu et al., Nanoconfined Co_3_O_4_ in hollow carbon spheres unlocks synergistic nonradical pathways for superior persulfate activation. Chem. Eng. J. **526**, 171344 (2025). 10.1016/j.cej.2025.171344

[18] Y. Gao, J. Zhang, H. Jin, G. Liang, L. Ma et al., Regenerating spent graphite from scrapped lithium-ion battery by high-temperature treatment. Carbon **189**, 493–502 (2022). 10.1016/j.carbon.2021.12.053

[19] Z. Ren, H. Li, W. Yan, W. Lv, G. Zhang et al., Comprehensive evaluation on production and recycling of lithium-ion batteries: a critical review. Renew. Sustain. Energy Rev. **185**, 113585 (2023). 10.1016/j.rser.2023.113585

[20] T. Ouaneche, L. Stievano, L. Monconduit, C. Guéry, M.T. Sougrati et al., The art of lithiation revisited: solvent-free room temperature reaction. Energy Storage Mater. **70**, 103507 (2024). 10.1016/j.ensm.2024.103507

[21] Z. Wang, Z. Li, J. Zhong, B. Zhou, J. Liu et al., A low-temperature solid-to-solid reaction for lithium-ion battery recycling and the utilization of defect-enriched Co_3_O_4_ from spent LiCoO_2_ batteries for efficient oxygen evolution reaction. Appl. Catal. B Environ. Energy **349**, 123873 (2024). 10.1016/j.apcatb.2024.123873

[22] G. Chen, B. Yuan, J. Dang, L. Xia, C. Zhang et al., Recycling the spent LiNi_1__−__*x*__*−*__*y*_Mn_*x*_CoyO2 cathodes for high-performance electrocatalysts toward both the oxygen catalytic and methanol oxidation reactions. Small **20**(15), 2306967 (2024). 10.1002/smll.202306967 10.1002/smll.202306967 37992250

[23] M. Shan, S. Xu, Y. Cao, B. Han, X. Zhu et al., Rapid regeneration of graphite anodes via self-induced microwave plasma. Adv. Funct. Mater. **34**(48), 2411834 (2024). 10.1002/adfm.202411834

[24] Y. Zhang, Z. Li, H. Jang, M.G. Kim, J. Cho et al., In situ grown RuNi alloy on ZrNiN_*x*_ as a bifunctional electrocatalyst boosts industrial water splitting. Adv. Mater. **37**(16), e2501586 (2025). 10.1002/adma.202501586 40052632 10.1002/adma.202501586

[25] H. Shi, T.-Y. Dai, X.-Y. Sun, Z.-L. Zhou, S.-P. Zeng et al., Dual-intermetallic heterostructure on hierarchical nanoporous metal for highly efficient alkaline hydrogen electrocatalysis. Adv. Mater. **36**(38), 2406711 (2024). 10.1002/adma.202406711 10.1002/adma.202406711 39046064

[26] G. Ding, J. Zhang, D. Yan, Y. Yu, L. Shuai et al., High-entropy regulation of lattice oxygen p-band toward sustainable electrocatalytic biomass valorization. Nano Lett. **25**(22), 8984–8992 (2025). 10.1021/acs.nanolett.5c01259 40407365 10.1021/acs.nanolett.5c01259

[27] X. Li, M. Chen, Y. Ye, C. Chen, Z. Li et al., Electronic structure modulation of nickel sites by cationic heterostructures to optimize ethanol electrooxidation activity in alkaline solution. Small **19**(18), e2207086 (2023). 10.1002/smll.202207086 36650993 10.1002/smll.202207086

[28] P.A. Shinde, V. Mahamiya, M. Safarkhani, N.R. Chodankar, M. Ishii et al., Unveiling the nanoarchitectonics of interfacial electronic coupling in atomically thin 2D WO_3_/WSe_2_ heterostructure for sodium-ion storage in aqueous system. Adv. Funct. Mater. **34**(41), 2406333 (2024). 10.1002/adfm.202406333

[29] J. Zhang, Y. Yang, G. Ding, Z. Wang, P. Wang et al., Electrooxidation of biomass-derived 5-hydroxymethylfurfural over sulfur-doped nickel–iron layered double hydroxides nanosheets. Chem. Eng. J. **505**, 159165 (2025). 10.1016/j.cej.2024.159165

[30] J. Zhang, D. Yan, G. Ding, X. Wang, C. Li et al., Dual co sites in n─n type heterojunction enable selective electrochemical co-valorization of HMF and CO_2_. Angew. Chem. Int. Ed. **64**(37), e202511448 (2025). 10.1002/anie.202511448 10.1002/anie.202511448 40641010

[31] D. Malhotra, D.T. Tran, S. Prabhakaran, D.H. Kim, N.H. Kim et al., Heterogeneous interface mismatch-manipulated ruthenium-immobilized binary metal phosphide-layered 2D V_2_CT_*x*_ for high-efficiency water electrolysis. Chem. Eng. J. **512**, 162665 (2025). 10.1016/j.cej.2025.162665

[32] N. Manivelan, J. Piao, J. Kim, S. Lee, Y. Kim et al., Unveiling the aluminum doping effects of in-situ transmogrified dual-LDH heterostructure and its Fermi-level alignment to water splitting potentials. Adv. Energy Mater. **15**(14), 2403889 (2025). 10.1002/aenm.202403889

[33] Y. Zhang, J. Liu, Y. Xu, C. Xie, S. Wang et al., Design and regulation of defective electrocatalysts. Chem. Soc. Rev. **53**(21), 10620–10659 (2024). 10.1039/d4cs00217b 39268976 10.1039/d4cs00217b

[34] Y. Zeng, M. Zhao, Z. Huang, W. Zhu, J. Zheng et al., Surface reconstruction of water splitting electrocatalysts. Adv. Energy Mater. **12**(33), 2201713 (2022). 10.1002/aenm.202201713

[35] Z.-Q. Ge, J. Li, H.-J. Zhang, C. Liu, G. Che et al., P–d orbitals coupling heterosites of Ni_2_P/NiFe-LDH interface enable O─H cleavage for water splitting. Adv. Funct. Mater. **34**(40), 2411024 (2024). 10.1002/adfm.202411024

[36] X. Li, T. Wu, N. Li, S. Zhang, W. Chang et al., Vertically staggered porous Ni_2_P/Fe_2_P nanosheets with trace Ru doping as bifunctional electrocatalyst for alkaline seawater splitting. Adv. Funct. Mater. **34**(34), 2400734 (2024). 10.1002/adfm.202400734

[37] W. Luo, Y. Yu, Y. Wu, Z. Ma, X. Ma et al., Realizing efficient oxygen evolution at low overpotential via dopant-induced interfacial coupling enhancement effect. Appl. Catal. B Environ. **336**, 122928 (2023). 10.1016/j.apcatb.2023.122928

[38] J.N. Hausmann, B. Traynor, R.J. Myers, M. Driess, P.W. Menezes, The pH of aqueous NaOH/KOH solutions: a critical and non-trivial parameter for electrocatalysis. ACS Energy Lett. **6**(10), 3567–3571 (2021). 10.1021/acsenergylett.1c01693

[39] B. Zhang, Y. Xu, B. Makuza, F. Zhu, H. Wang et al., Selective lithium extraction and regeneration of LiCoO_2_ cathode materials from the spent lithium-ion battery. Chem. Eng. J. **452**, 139258 (2023). 10.1016/j.cej.2022.139258

[40] V.S. Sikarwar, M. Hrabovský, G. Van Oost, M. Pohořelý, M. Jeremiáš, Progress in waste utilization via thermal plasma. Prog. Energy Combust. Sci. **81**, 100873 (2020). 10.1016/j.pecs.2020.100873

[41] J. Lv, L. Wang, R. Li, K. Zhang, D. Zhao et al., Constructing a hetero-interface composed of oxygen vacancy-enriched Co_3_O_4_ and crystalline-amorphous NiFe-LDH for oxygen evolution reaction. ACS Catal. **11**(23), 14338–14351 (2021). 10.1021/acscatal.1c03960

[42] Z. Liu, H. Yuan, Z. Wan, Z. Ma, X. Deng et al., Nanostructured Co_3_O_4_@NiFe-LDH heterojunction catalysts for improving oxygen evolution reaction in alkaline environment. J. Alloys Compd. **983**, 173837 (2024). 10.1016/j.jallcom.2024.173837

[43] B. Wang, X. Chen, Y. He, Q. Liu, X. Zhang et al., Fe_2_O_3_/P-doped CoMoO_4_ electrocatalyst delivers efficient overall water splitting in alkaline media. Appl. Catal. B Environ. **346**, 123741 (2024). 10.1016/j.apcatb.2024.123741

[44] D. Li, D. Xu, Y. Pei, Q. Zhang, Y. Lu et al., Isolated octahedral Pt-induced electron transfer to ultralow-content ruthenium-doped spinel Co_3_O_4_ for enhanced acidic overall water splitting. J. Am. Chem. Soc. **146**(42), 28728–28738 (2024). 10.1021/jacs.4c07089 39268752 10.1021/jacs.4c07089

[45] S. Nagappan, H. Gurusamy, H. Minhas, A. Karmakar, S. Ravichandran et al., Unraveling the synergistic role of Sm^3+^ doped NiFe-LDH as high-performance electrocatalysts for improved anion exchange membrane and water splitting applications. Small Methods **9**(5), 2401655 (2025). 10.1002/smtd.202401655 10.1002/smtd.202401655 39686802

[46] S. Ye, W. Chen, Z. Ou, Q. Zhang, J. Zhang et al., Harnessing the synergistic interplay between atomic-scale vacancies and ligand effect to optimize the oxygen reduction activity and tolerance performance. Angew. Chem. Int. Ed. **64**(2), e202414989 (2025). 10.1002/anie.202414989 10.1002/anie.202414989 39233354

[47] R. Wang, X. Sun, J. Zhong, S. Wu, Q. Wang et al., Low-temperature plasma-assisted synthesis of iron and nitrogen Co-doped CoFeP-N nanowires for high-efficiency electrocatalytic water splitting. Appl. Catal. B Environ. **352**, 124027 (2024). 10.1016/j.apcatb.2024.124027

[48] S. Zhang, C. Tan, R. Yan, X. Zou, F.-L. Hu et al., Constructing built-in electric field in heterogeneous nanowire arrays for efficient overall water electrolysis. Angew. Chem. Int. Ed. **62**(26), e202302795 (2023). 10.1002/anie.202302795 10.1002/anie.202302795 37046392

[49] L. Chong, J. Wen, E. Song, Z. Yang, I.D. Bloom et al., Synergistic Co─Ir/Ru composite electrocatalysts impart efficient and durable oxygen evolution catalysis in acid. Adv. Energy Mater. **13**(37), 2302306 (2023). 10.1002/aenm.202302306

[50] X. Teng, Z. Wang, Y. Wu, Y. Zhang, B. Yuan et al., Enhanced alkaline hydrogen evolution reaction of MoO_2_/Ni_3_S_2_ nanorod arrays by interface engineering. Nano Energy **122**, 109299 (2024). 10.1016/j.nanoen.2024.109299

[51] Y. Shi, L. Song, Y. Liu, T. Wang, C. Li et al., Dual cocatalytic sites synergize NiFe layered double hydroxide to boost oxygen evolution reaction in anion exchange membrane water electrolyzer. Adv. Energy Mater. **14**(46), 2402046 (2024). 10.1002/aenm.202402046

[52] T. Zhao, B. Gong, G. Xu, J. Jiang, L. Zhang, in situ surface reconstruction of heterostructure Ni_2_P/CoP/FeP_4_ nanowires network catalyst for high-current-density overall water splitting. Chin. J. Catal. **61**, 269–280 (2024). 10.1016/S1872-2067(24)60037-9

[53] H. Liu, Z. Li, J. Hu, Z. Qiu, W. Liu et al., Self-supported cobalt oxide electrocatalysts with hierarchical chestnut burr-like nanostructure for efficient overall water splitting. Chem. Eng. J. **435**, 134995 (2022). 10.1016/j.cej.2022.134995

[54] J. Zhang, X. Zhang, Z. Ma, K. Fang, L. Wang et al., POM-intercalated NiFe-LDH as enhanced OER catalyst for highly efficient and durable water electrolysis at ampere-scale current densities. ACS Catal. **15**(8), 6486–6496 (2025). 10.1021/acscatal.5c00448

[55] X. Liu, Q. Yu, X. Qu, X. Wang, J. Chi et al., Manipulating electron redistribution in Ni_2_P for enhanced alkaline seawater electrolysis. Adv. Mater. **36**(1), e2307395 (2024). 10.1002/adma.202307395 37740701 10.1002/adma.202307395

