# Supplementary material for: Directional Reconstruction of Spent Lithium Cobalt Oxide by Microwave Plasma: Efficient Oxygen Evolution Catalyst from Closed‑Loop Recovered Resources
Source: Nanomicro Lett. 2026 Apr 13;18:327. doi: 10.1007/s40820-026-02164-1 (PMC13076869; doi:10.1007/s40820-026-02164-1)
Supplement: Supplementary file 1 — Supplementary file1 (DOCX 39366 kb) [file 40820_2026_2164_MOESM1_ESM.docx]

Supporting Information for

**Directional Reconstruction of Spent Lithium Cobalt Oxide by Microwave Plasma: Efficient Oxygen Evolution Catalyst from Closed-Loop Recovered Resources**

Chao Chen^1,2^, Qixuan Zhu^2^, Minghui Shan^2^, Lei Cheng^2^*,* Weiwei Wang^2^, Zhiqiang Lu^2^, Yi Wang^2^, Hengda Sun^1,2^, Yusuke Yamauchi^4, 5, 6^, Jing Tang^3^*, Guiyin Xu^1,2^*

^1^ Henan Academy of Sciences, School of Materials Science and Engineering, Zhengzhou University, Henan 450052, P. R. China

^2^ State Key Laboratory of Advanced Fiber Materials, College of Materials Science and Engineering, Donghua University, Shanghai 201620, P. R. China

^3^ State Key Laboratory of Petroleum Molecular and Process Engineering, Shanghai Key Laboratory of Green Chemistry and Chemical Processes, School of Chemistry and Molecular Engineering, East China Normal University, Shanghai 200062, P. R. China

^4^ Australian Institute for Bioengineering and Nanotechnology (AIBN) and School of Chemical Engineering, The University of Queensland, Brisbane, Queensland 4000, Australia

^5^ Department of Materials Process Engineering, Graduate School of Engineering, Nagoya University, Nagoya, Japan

^6^ Department of Chemical and Biomolecular Engineering, Yonsei University, 50 Yonsei-ro, Seodaemun-gu, Seoul 03722, South Korea

*Corresponding authors. E-mail: jingtang@chem.ecnu.edu.cn (Jing Tang); xuguiyin@dhu.edu.cn (Guiyin Xu)

**Supplementary Figures and Tables**


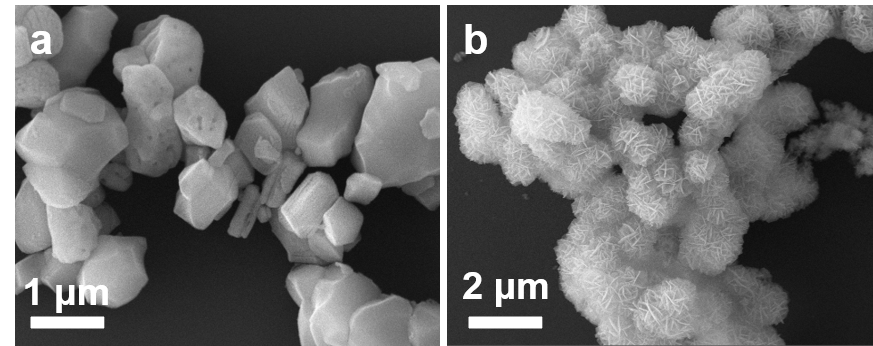

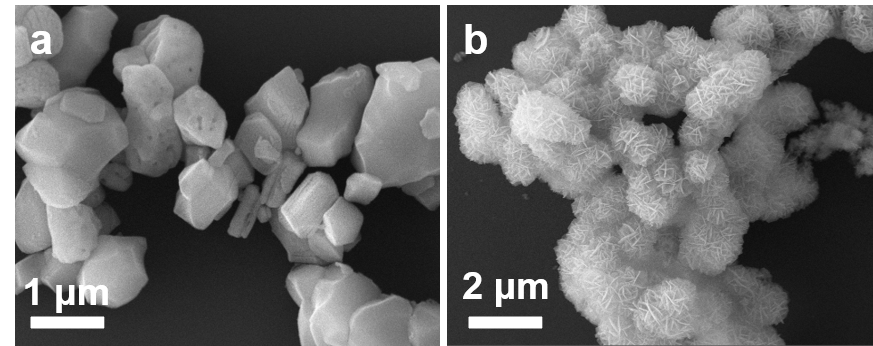


**Fig. S1 a** SEM images of Co_3_O_4_. **b** SEM images of NiFe-LDH

**Fig. S2 a** HRTEM images of NiFe-LDH. **b** HRTEM images of Co_3_O_4_/NiFe-LDH

**
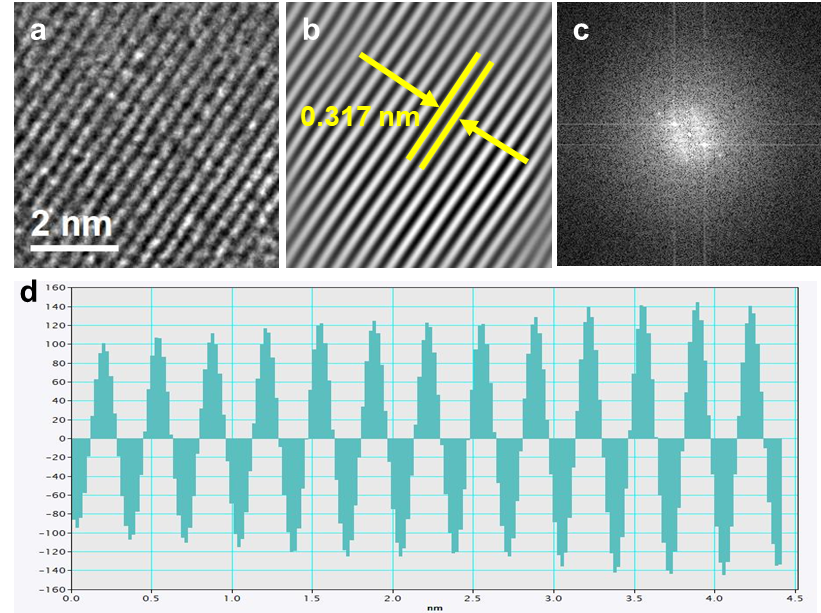
**

**Fig. S3** **a** HRTEM images of (110) crystal planes. **b** Corresponding IFFT patterns and lattice distance of a. **c** Corresponding FFT patterns of the selected regions marked of a. **d** The corresponding lattice spacing distances transferred by fast Fourier transform

**
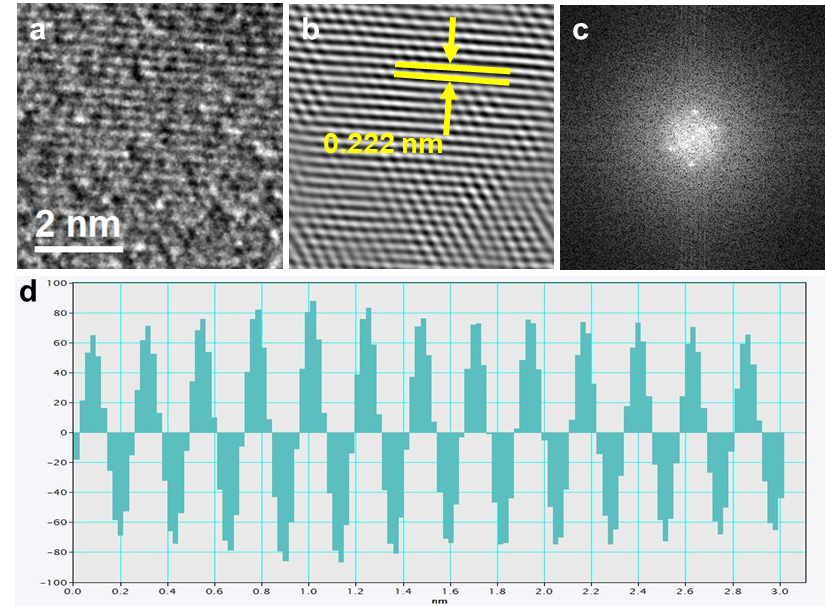
**

**Fig. S4 a** HRTEM images of (311) crystal planes. **b** Corresponding IFFT patterns and lattice distance of a. **c** Corresponding FFT patterns of the selected regions marked of a. **d** The corresponding lattice spacing distances transferred by fast Fourier transform

**
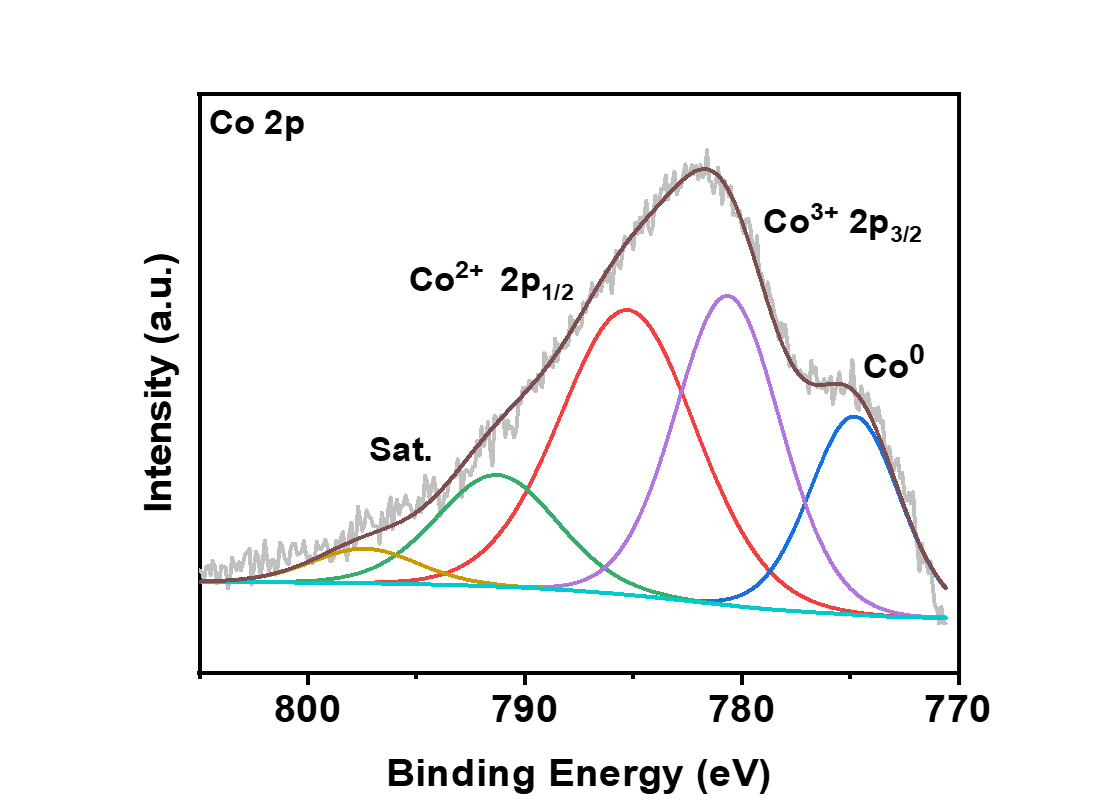
**

**Fig. S5** XPS spectra of Co 2p in Co_3_O_4_/NiFe-LDH (Co^2+^ 785.3 eV, Co^3+^ 780.6 eV)


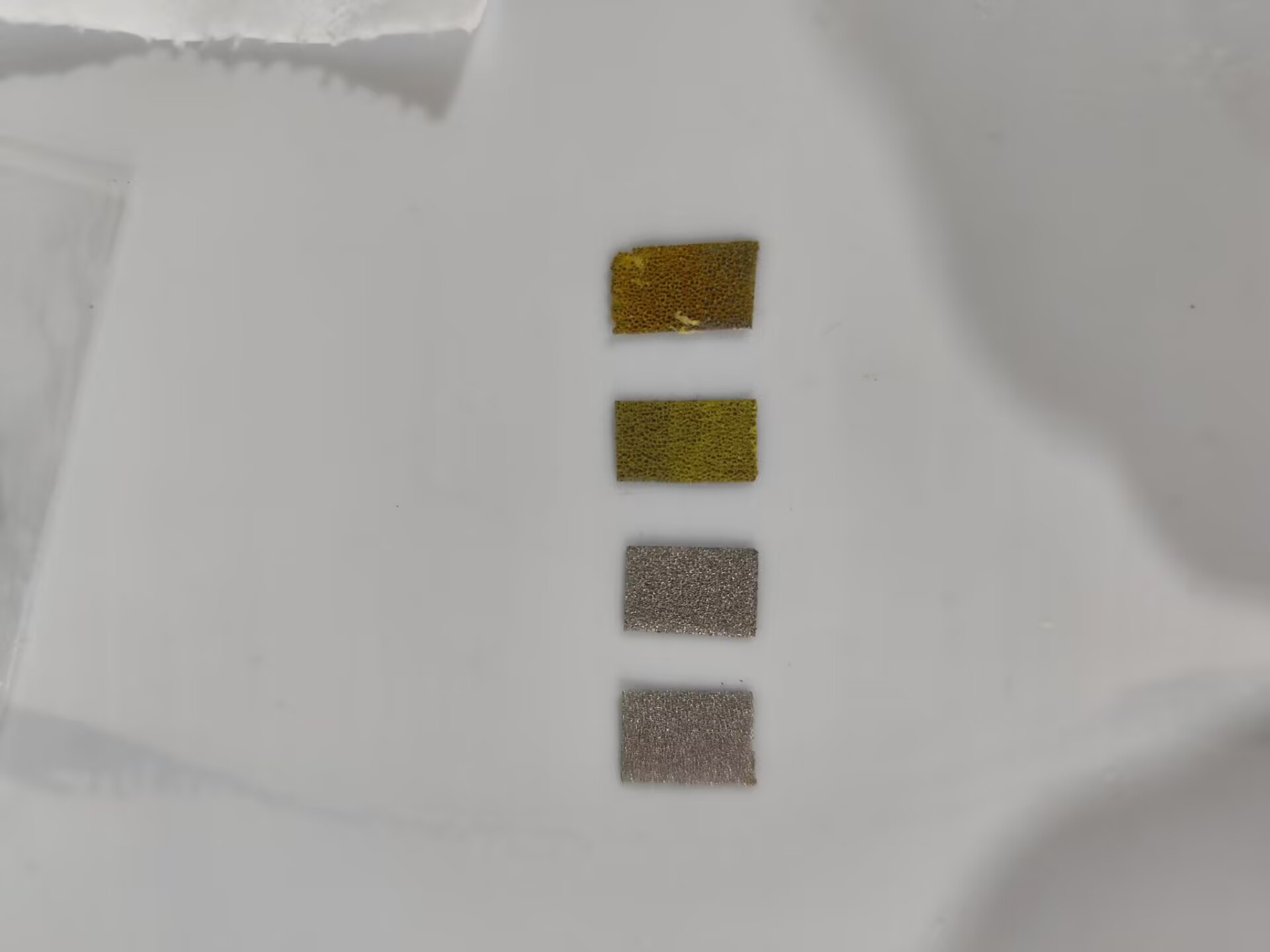


**Fig. S6** Digital photos of bare NF, treated NF, NiFe-LDH and Co_3_O_4_/NiFe-LDH

**Fig. S7** Cyclic voltammogram (CV) curves of Co_3_O_4_/NiFe-LDH

**Fig. S8** Linear sweep voltammetry (LSV) curves of Co_3_O_4_/NiFe-LDH and Co_3_O_4_+NiFe-LDH-mix

**
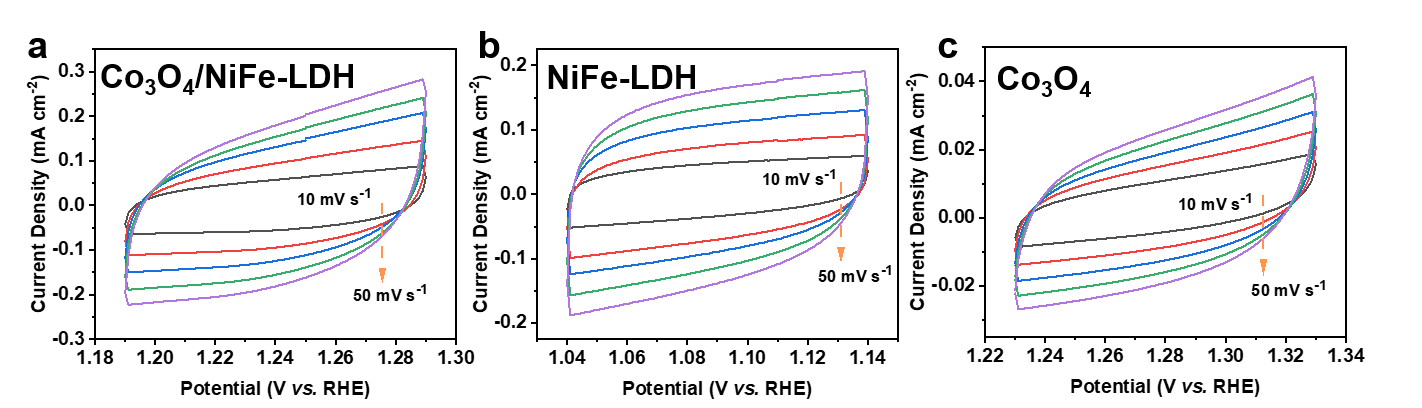
**

**
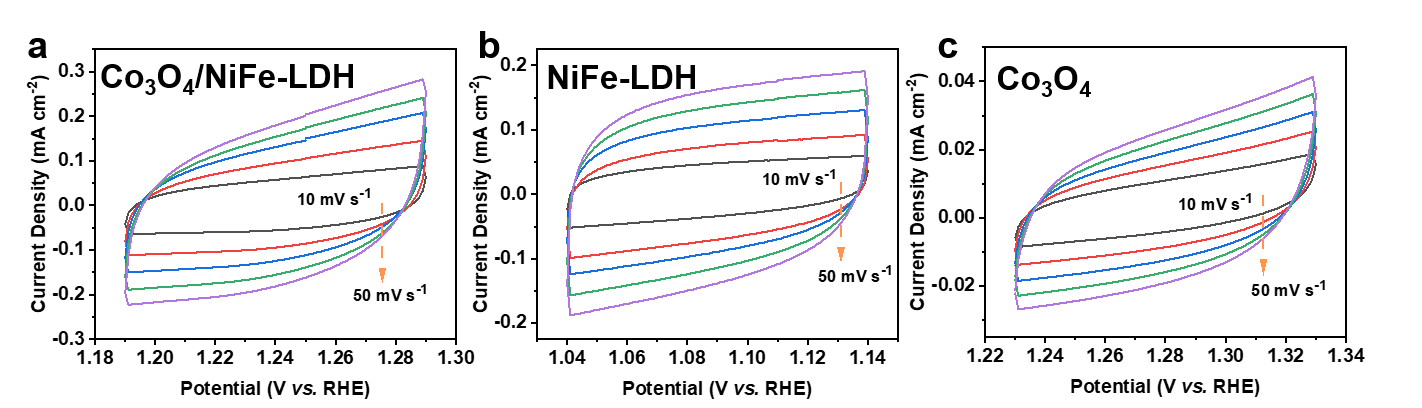
**

**
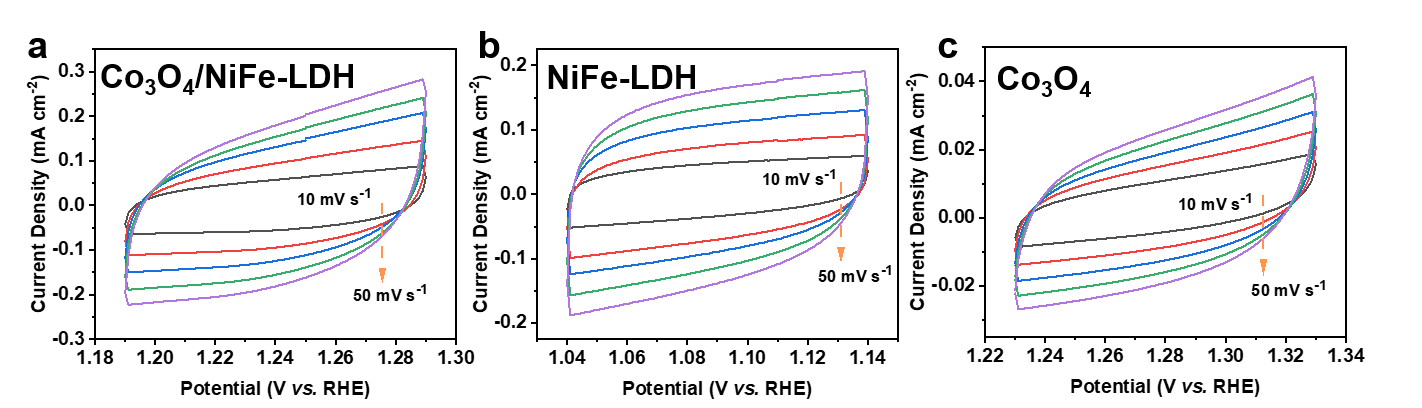
**

**Fig. S9** Cyclic voltammogram (CV) curves of **a** Co_3_O_4_/NiFe-LDH. **b** NiFe-LDH. **c** Co_3_O_4_ at different scan rates (10 ~ 50 mV s^-1^) in alkaline media

**Fig. S10** ECSA-normalized polarization curves of Co_3_O_4_/NiFe-LDH and NiFe-LDH

**Fig. S11** SEM image of Co_3_O_4_/NiFe-LDH after the test

**Fig. S12** Elemental mappings of Co_3_O_4_/NiFe-LDH catalysts after the test

**Fig. S13** FTIR spectra of Co_3_O_4_/NiFe-LDH before and after catalytic reaction

**Fig. S14** Stability test of the IrO_2_ for OER at 10 mA cm^−2^ in 1.0 M KOH


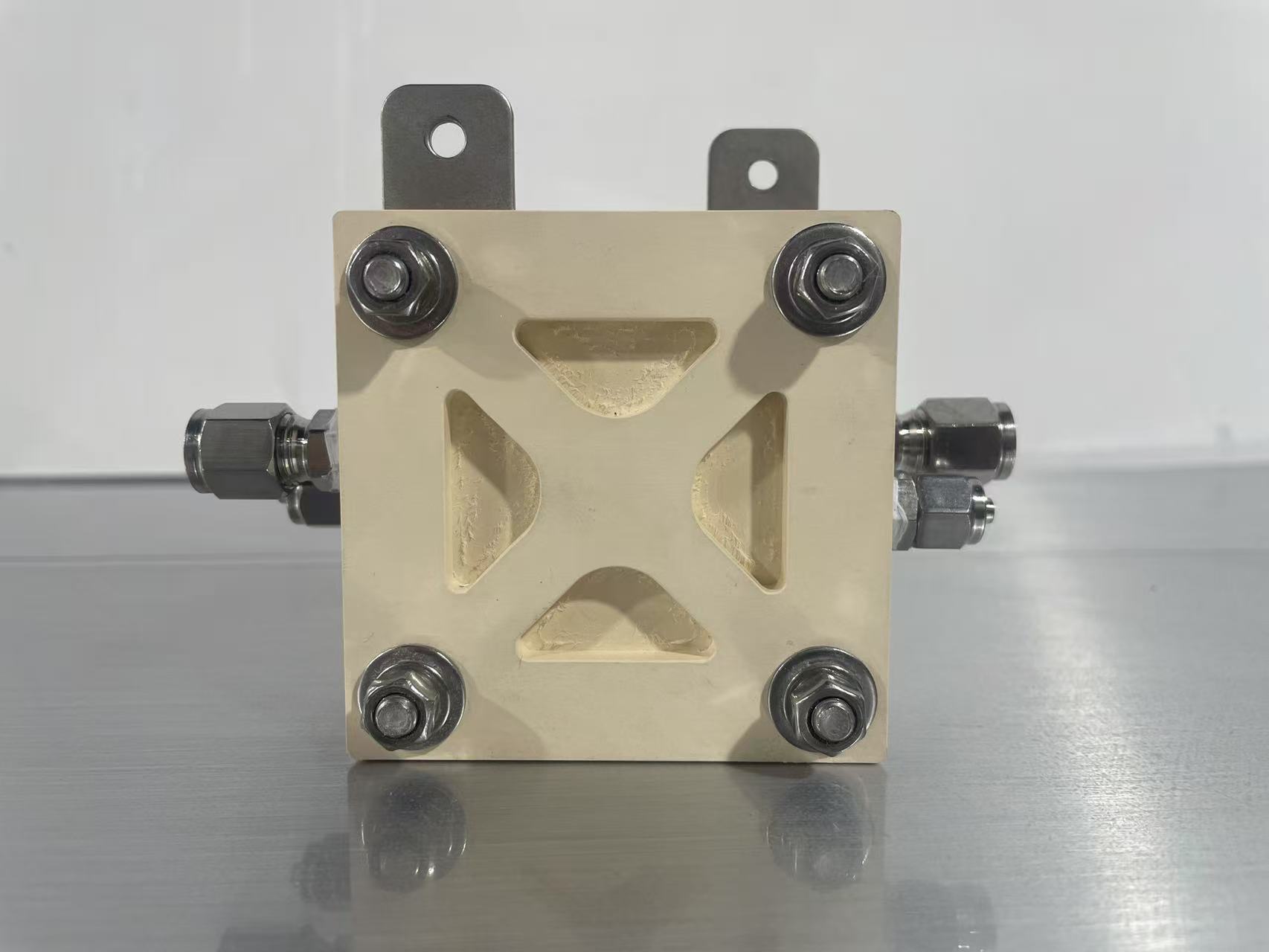


**Fig. S15** Digital photos of electrolytic cell


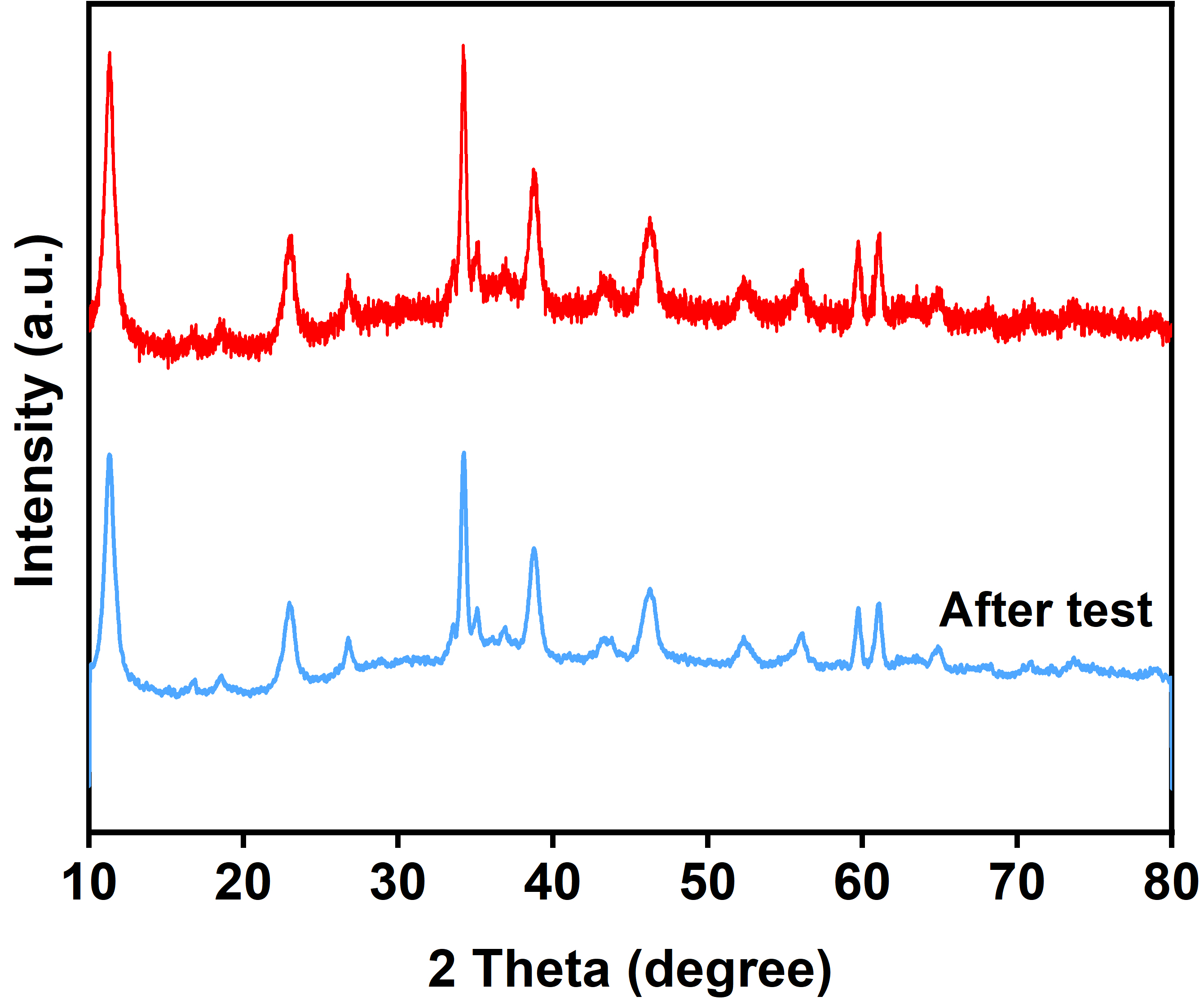


**Fig. S16** XRD spectra of Co_3_O_4_/NiFe-LDH before and after catalytic reaction


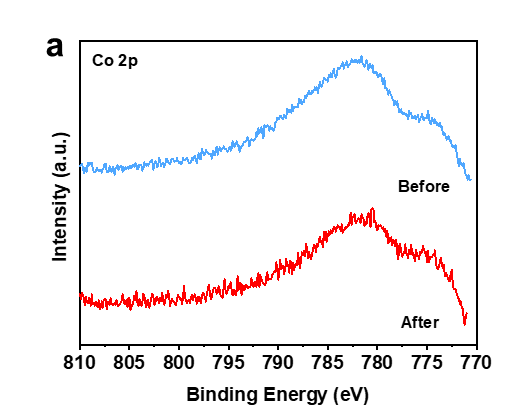

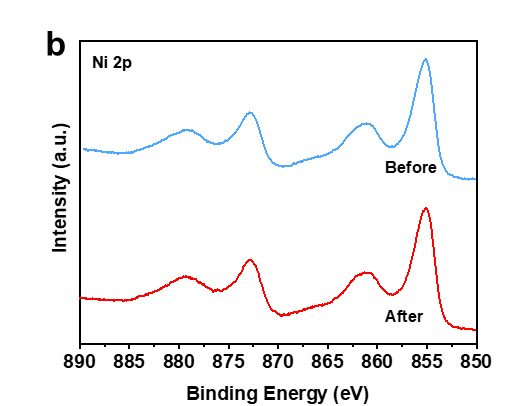

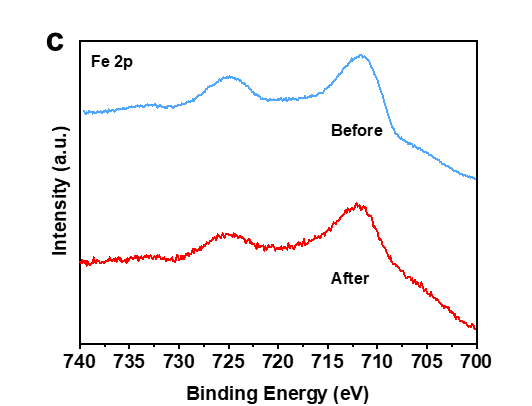


**Fig. S17** High-resolution **a** Co 2p. **b** Ni 2p and **c** Fe 2p XPS spectra of Co_3_O_4_/NiFe-LDH before and after the stability test

**Fig. S18** The stability test of Co_3_O_4_/NiFe-LDH in 30 wt% KOH at 500 mA cm^−2^


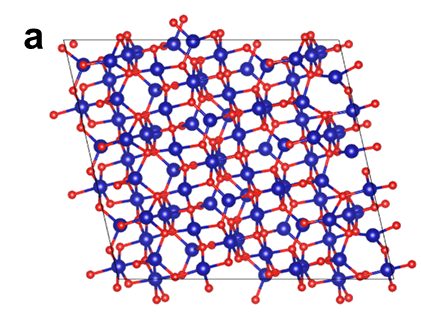


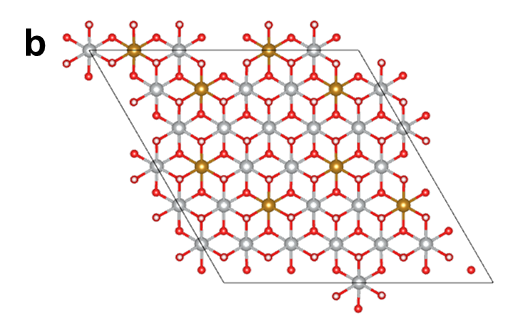


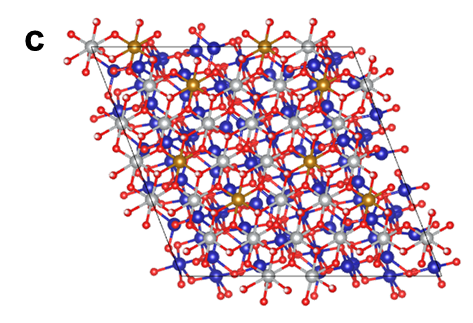


**Fig. S19** Optimal crystal structural models of **a** Co_3_O_4._ **b** NiFe-LDH. **c** Co_3_O_4_/NiFe-LDH. The blue, gray and yellow spheres represent Co, Ni and Fe atoms, respectively


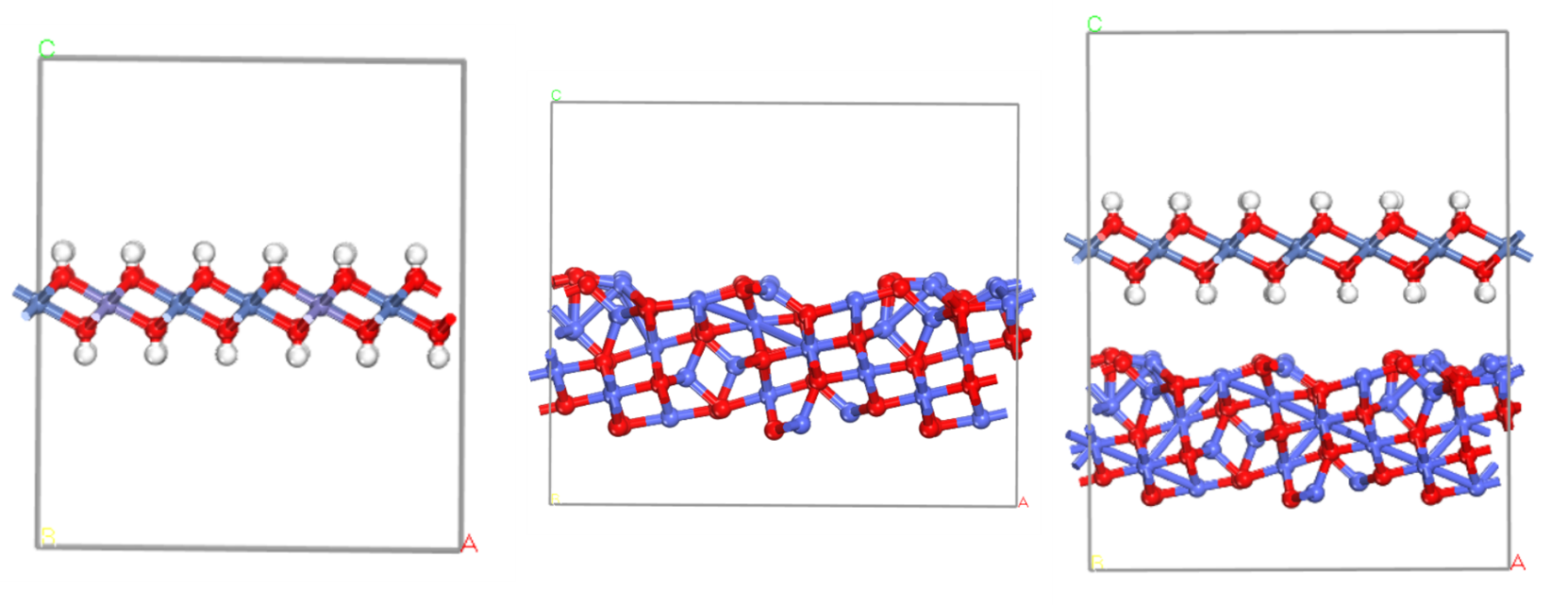


**a**


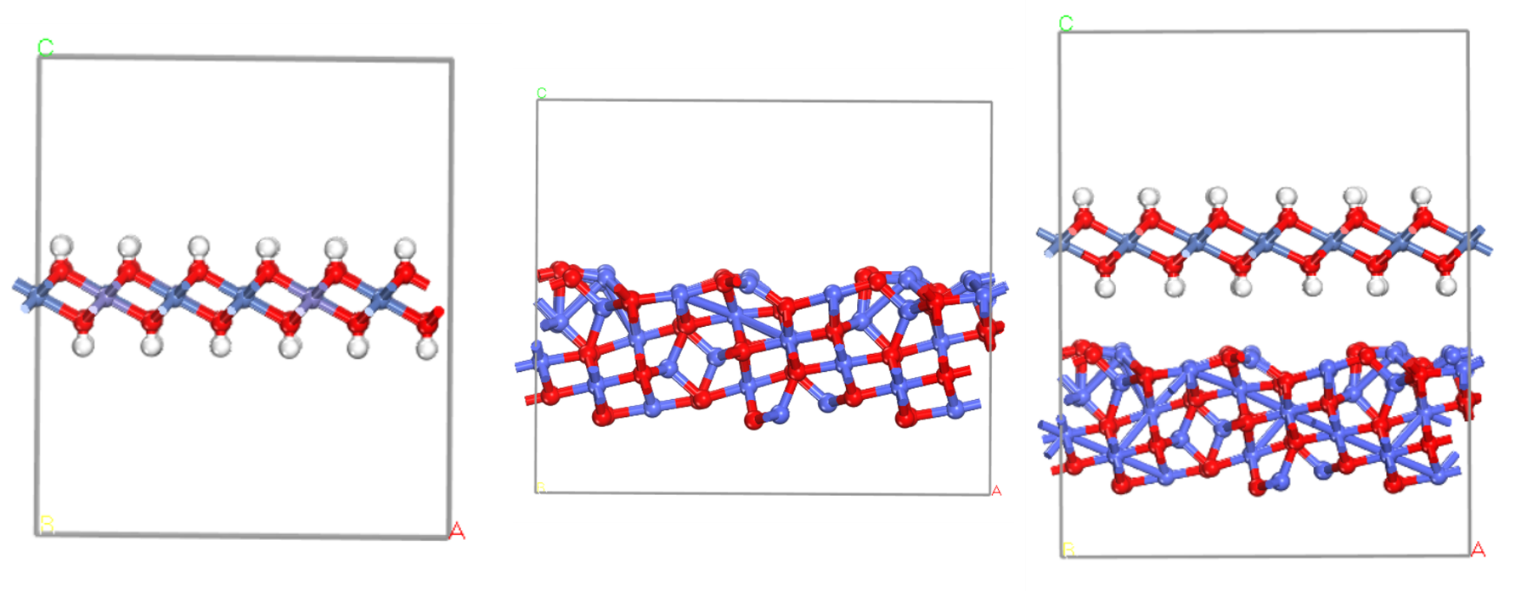


**b**


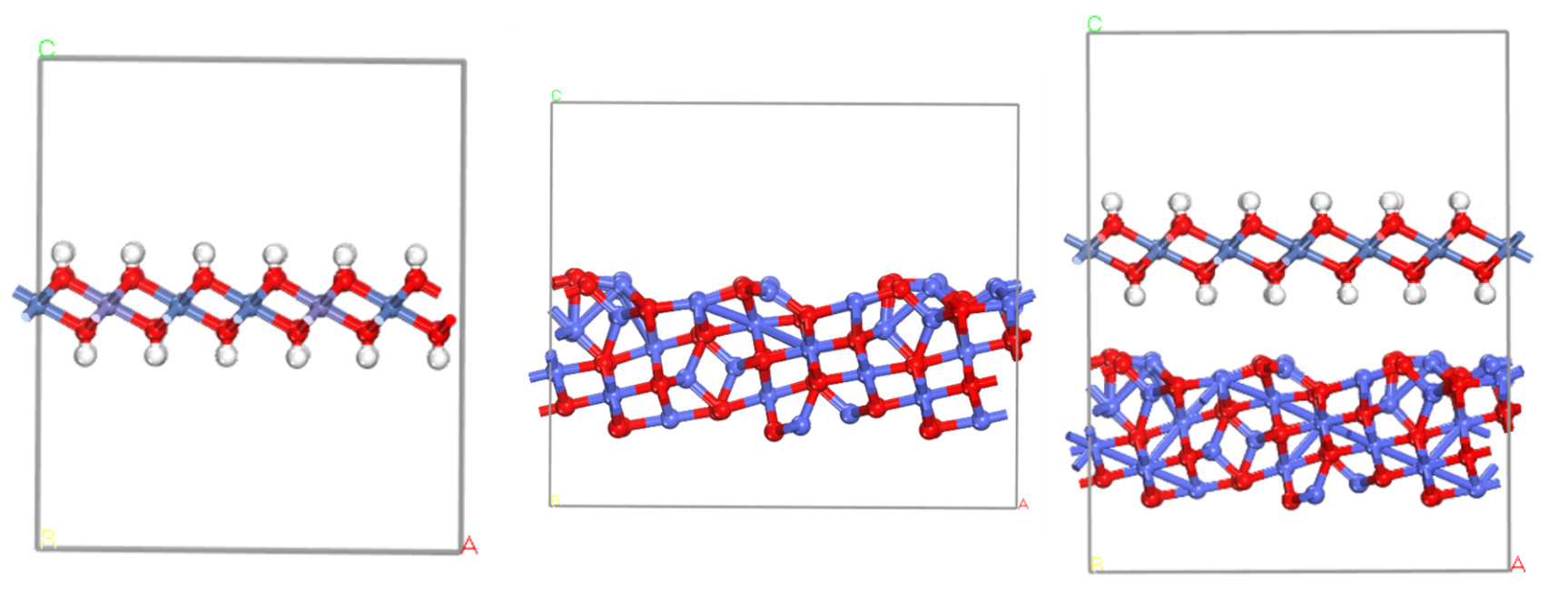


**c**

**Fig. S20** Optimized optimal crystal structural models of **a** Co_3_O_4_. **b** NiFe-LDH. **c** Co_3_O_4_/NiFe-LDH


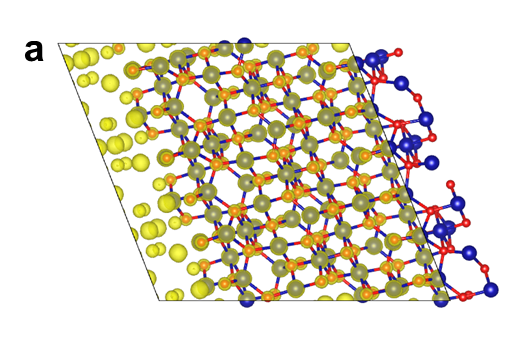

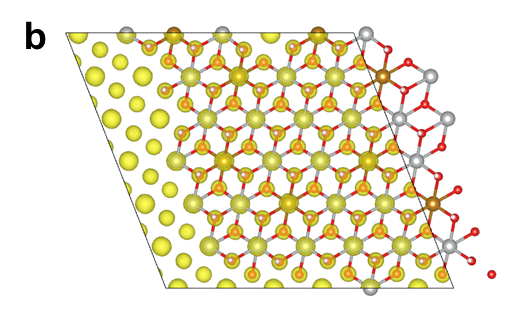


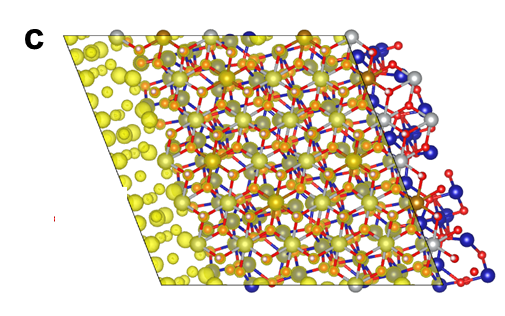


**Fig. S21** Charge density difference analysis. Yellow isosurfaces represent regions of electron accumulation. (Isosurface value: 0.1-1)

**Table S1** The element content of Co_3_O_4_/NiFe-LDH catalysts determined from ICP-OES results

| Element | ppm | molar ratios |
| --- | --- | --- |
| Co | 3.03 | 1 |
| Ni | 31.9 | 10.58 |
| Fe | 9.67 | 3.37 |

**Table S2** The element content of Co_3_O_4_/NiFe-LDH catalysts determined from EDS mapping

| Sample | Co/wt% | Ni/ wt% | Fe/ wt% | O/ wt% |
| --- | --- | --- | --- | --- |
| Co_3_O_4_/NiFe-LDH | 5.11 | 49.23 | 9.68 | 35.99 |
| After test | 5.01 | 49.03 | 9.60 | 36.37 |

**Table S3** Comparisons of OER performance of various electrocatalysts in 1.0 M KOH

| Sample | *E* _η10_  (mV) | *E* _η10-noble metal_  (mV) | References |
| --- | --- | --- | --- |
| Co_3_O_4_/NiFe-LDH | 235 | 306 (IrO_2_) | This work |
| Co_3_O_4_/NiFe-LDH-2 | 238 | 306 (IrO_2_) | This work |
| WQ-Co_3_O_4_-400 | 306 | 309 (RuO_2_) | [S1] |
| Fe-Co_3_O_4_ NBs | 254 | 292 (IrO_2_) | [S2] |
| CoFeV | 266 | ~280 (IrO_2_) | [S3] |
| NdNi-Co_3_O_4_ | 269 | 292 (RuO_2_) | [S4] |
| Co_3_O_4_@Fe_1_-NC | 318 | -- | [S5] |
| W-Co_3_O_4_ | 390 | -- | [S6] |
| Co/Co_3_O_4_/CoF_2_@NSC-CC | 310 | 340 (RuO_2_) | [S7] |
| Co_3_O_4_-V_Co_ | 262 | -- | [S8] |
| Co_3_C | 330 | -- | [S9] |
| RCO-V_O_@CC | 253 | -- | [S10] |
| CoFe-FeNC | 296 | 330 (IrO_2_) | [S11] |
| NiCoS | 248 | 321 (RuO_2_) | [S12] |
| ELFP-Ni | 250 | 290 (RuO_2_) | [S13] |
| Fe-LCO/Co_3_O_4_ | 312 | 325 (IrO_2_) | [S14] |
| NCO@7CQDs | 330 | 517 (RuO_2_) | [S15] |
| FCN-TM/NC | 270 | 314 (RuO_2_) | [S16] |

**Table S4** Comparison of our work with recently reported OER catalysts for AWE electrolyzers in 30 wt% KOH

| **Cathode//Anode Catalysts** | **Temperature**  **(°C)** | **Area (cm^2^)** | **Voltage (V)** | **References** |
| --- | --- | --- | --- | --- |
| **NF//Co_3_O_4_/NiFe-LDH** | **80** | **1** | **1.74 @ 0.2 A cm^-2^** | **This work** |
| NF//Ni(OH)_2_ | N/A | N/A | 2.05 @ 1 A cm^-2^ | [S17] |
| Ni(OH)_2_TPE //Ni(OH)_2_TPE | 80 | N/A | 1.8 @ 0.91 A cm^-2^ | [S18] |
| Ni_3_S_2_ /NM-I//Ni_3_S_2_/NM-I | 80 | 4 | 1.8 @ 1 A cm^-2^ | [S19] |
| Co,P-MoS_2_@NiO// Co,P-MoS_2_@NiO | 60 | N/A | 1.82 @ 1 A cm^-2^ | [S20] |
| R-Ni//FeNiHOF | 60 | N/A | 1.81 @1 A cm^-2^ | [S21] |
| WMo-CoP@NM//NM | 85 | N/A | 2 @1.25 A cm^-2^ | [S22] |

**Supplementary References**

1. Z. Wang, Z. Li, J. Zhong, B. Zhou, J. Liu et al., A low-temperature solid-to-solid reaction for lithium-ion battery recycling and the utilization of defect-enriched Co_3_O_4_ from spent LiCoO_2_ batteries for efficient oxygen evolution reaction. Appl. Catal. B Environ. Energy **349**, 123873 (2024). <https://doi.org/10.1016/j.apcatb.2024.123873>
2. D. Zhao, G.-Q. Yu, J. Xu, Q. Wu, W. Zhou et al., Preparing iron oxide clusters surface modified Co_3_O_4_ nanoboxes by chemical vapor deposition as an efficient electrocatalyst for oxygen evolution reaction. Energy Storage Mater. **66**, 103236 (2024). <https://doi.org/10.1016/j.ensm.2024.103236>
3. K. Yeom, J. Jo, H. Shin, H. Ji, S. Moon et al., Unraveling surface reconstruction during oxygen evolution reaction on the defined spinel oxide surface. Adv. Funct. Mater. **34**(36), 2401095 (2024). <https://doi.org/10.1002/adfm.202401095>
4. T. Li, Z. Wang, L. Wang, M. Wang, Y.-Q. Liu, Nd and Ni Co-doped spinel Co_3_O_4_ nanosheet as an effective electrocatalyst for oxygen evolution reaction. Appl. Catal. B Environ. Energy **352**, 123990 (2024). <https://doi.org/10.1016/j.apcatb.2024.123990>
5. Y. Xie, Y. Feng, S. Zhu, Y. Yu, H. Bao et al., Modulation in spin state of Co_3_O_4_ decorated Fe single atom enables a superior rechargeable zinc-air battery performance. Adv. Mater. **37**(5), 2414801 (2025). <https://doi.org/10.1002/adma.202414801>
6. T. Tran-Phu, M. Chatti, J. Leverett, T.K.A. Nguyen, D. Simondson et al., Understanding the role of (W, Mo, Sb) dopants in the catalyst evolution and activity enhancement of Co_3_O_4_ during water electrolysis *via* *in situ* spectroelectrochemical techniques. Small **19**(25), e2208074 (2023). <https://doi.org/10.1002/smll.202208074>
7. H. Li, G. Yan, H. Zhao, P.C. Howlett, X. Wang et al., Earthworm-inspired Co/Co_3_O_4_/CoF_2_@NSC nanofibrous electrocatalyst with confined channels for enhanced ORR/OER performance. Adv. Mater. **36**(26), 2311272 (2024). <https://doi.org/10.1002/adma.202311272>
8. R. Zhang, L. Pan, B. Guo, Z.-F. Huang, Z. Chen et al., Tracking the role of defect types in Co_3_O_4_ structural evolution and active motifs during oxygen evolution reaction. J. Am. Chem. Soc. **145**(4), 2271–2281 (2023). <https://doi.org/10.1021/jacs.2c10515>
9. Y. Oh, J. Theerthagiri, A. Min, C.J. Moon, Y. Yu et al., Pulsed laser interference patterning of transition-metal carbides for stable alkaline water electrolysis kinetics. Carbon Energy **6**(5), e448 (2024). <https://doi.org/10.1002/cey2.448>
10. W. Tian, X. Xie, X. Zhang, J. Li, G.I.N. Waterhouse et al., Synergistic interfacial effect of Ru/Co_3_O_4_ heterojunctions for boosting overall water splitting. Small **20**(27), 2309633 (2024). <https://doi.org/10.1002/smll.202309633>
11. S. Zhang, J. Yang, L. Yang, T. Yang, Y. Liu et al., Boosting ORR/OER bifunctional electrocatalysis by promoting electronic redistribution of Fe-N-C on CoFe-FeNC for ultra-long rechargeable Zn-air batteries. Appl. Catal. B Environ. Energy **359**, 124485 (2024). <https://doi.org/10.1016/j.apcatb.2024.124485>
12. M. Chen, Y. Wang, Y. Zhou, B. Guo, L. Wang et al., Efficient metal recovery and electrocatalyst fabrication from spent lithium-ion batteries *via* green solvent extraction. Green Chem. **27**(18), 5126–5135 (2025). <https://doi.org/10.1039/d5gc00073d>
13. J. Liu, Z. Liu, Z. Xiao, Y. Zhu, J. Wang et al., Iodine-mediated redox strategy for sustainable lithium extraction from spent LiFePO_4_ cathodes. Adv. Mater. **37**(26), 2503450 (2025). <https://doi.org/10.1002/adma.202503450>
14. G. Zhang, C. Ye, T. Li, S. Liu, W.-H. Huang et al., Quenching-induced Fe doping on spent cathode materials enhances the oxygen evolution reaction performance. Energy Storage Mater. **80**, 104430 (2025). <https://doi.org/10.1016/j.ensm.2025.104430>
15. Q. Liang, Y. Liu, H. Kang, L. Zhou, C. Wang et al., Waste to wealth: Spent LIB anode graphite saved to carbon quantum dots strongly coupled NiCo_2_O_4_ catalysts for efficient water oxidation. Chem. Eng. J. **525**, 170376 (2025). <https://doi.org/10.1016/j.cej.2025.170376>
16. W. Liu, Z. Liang, S. Jing, J. Zhong, N. Liu et al., Asymmetrical triatomic sites with long-range electron coupling for ultra-durable and extreme-low-temperature zinc-air batteries. Angew. Chem. Int. Ed. **64**(21), e202503493 (2025). <https://doi.org/10.1002/anie.202503493>
17. J. Kang, G. Liu, Q. Hu, Y. Huang, L.-M. Liu et al., Parallel nanosheet arrays for industrial oxygen production. J. Am. Chem. Soc. **145**(46), 25143–25149 (2023). <https://doi.org/10.1021/jacs.3c05688>
18. W. Kuang, Z. Cui, C. Wang, T. Chen, Q. Wang et al., Self-supported Ni/Ni(OH)_2_ electrodes for high-performance alkaline and AEM water electrolysis. Adv. Energy Mater. **15**(14), 2406080 (2025). <https://doi.org/10.1002/aenm.202406080>
19. W. He, Y. Wang, Y. Zhao, C. Tang, L. Cong et al., Heterointerface-enabled anti-reverse-current electrodes for alkaline water electrolyzers at 1000 mA cm^-2^. J. Am. Chem. Soc. **148**(5), 5232–5242 (2026). <https://doi.org/10.1021/jacs.5c17603>
20. H. Li, J. Xia, J. Zheng, X. Bai, Q. Ren et al., Synergistic co and P co-doping in MoS_2_/NiO heterostructures for electronic modulation toward high-performance alkaline water electrolysis. Adv. Funct. Mater. e25383 (2025). <https://doi.org/10.1002/adfm.202525383>
21. Y. Chen, Q. Li, Y. Lin, J. Liu, J. Pan et al., Boosting oxygen evolution reaction by FeNi hydroxide-organic framework electrocatalyst toward alkaline water electrolyzer. Nat. Commun. **15**(1), 7278 (2024). <https://doi.org/10.1038/s41467-024-51521-4>
22. G. Xu, M. Xing, Z. Qiao, M. Han, Y. Wu et al., Constructing ultra-stable electrocatalysts to achieve adaptability of industrial-level alkaline water electrolyzers for fluctuating renewable energies. Adv. Energy Mater. **15**(31), 2500926 (2025). <https://doi.org/10.1002/aenm.202500926>
